# Dietary Probiotic Effect of *Lactococcus lactis* WFLU12 on Low-Molecular-Weight Metabolites and Growth of Olive Flounder (*Paralichythys olivaceus*)

**DOI:** 10.3389/fmicb.2018.02059

**Published:** 2018-09-05

**Authors:** Thanh Luan Nguyen, Won-Kyong Chun, Ahran Kim, Nameun Kim, Heyong Jin Roh, Yoonhang Lee, Myunggi Yi, Suhkmann Kim, Chan-Il Park, Do-Hyung Kim

**Affiliations:** ^1^Department of Veterinary Medicine, HUTECH Institute of Applied Science, Ho Chi Minh City University of Technology, Ho Chi Minh City, Vietnam; ^2^Department of Aquatic Life Medicine, College of Fisheries Science, Pukyong National University, Busan, South Korea; ^3^Department of Biomedical Engineering, College of Engineering, Pukyong National University, Busan, South Korea; ^4^Department of Chemistry, Center for Proteome Biophysics, Chemistry Institute for Functional Materials, Pusan National University, Busan, South Korea; ^5^Department of Marine Biology and Aquaculture, College of Marine Science, Gyeongsang National University, Tongyeong, South Korea

**Keywords:** probiotics, olive flounder, fish, growth promotion, metabolome, metabolite, CE-TOFMS

## Abstract

The use of probiotics is considered an attractive biocontrol method. It is effective in growth promotion in aquaculture. However, the mode of action of probiotics in fish in terms of growth promotion remains unclear. The objective of the present study was to investigate growth promotion effect of dietary administration of host-derived probiotics, *Lactococcus lactis* WFLU12, on olive flounder compared to control group fed with basal diet by analyzing their intestinal and serum metabolome using capillary electrophoresis mass spectrometry with time-of flight (CE-TOFMS). Results of CE-TOFMS revealed that 53 out of 200 metabolites from intestinal luminal metabolome and 5 out of 171 metabolites from serum metabolome, respectively, were present in significantly higher concentrations in the probiotic-fed group than those in the control group. Concentrations of metabolites such as citrulline, tricarboxylic acid cycle (TCA) intermediates, short chain fatty acids, vitamins, and taurine were significantly higher in the probiotic-fed group than those in the control group. The probiotic strain WFLU12 also possesses genes encoding enzymes to help produce these metabolites. Therefore, it is highly likely that these increased metabolites linked to growth promotion in olive flounder are due to supplementation of the probiotic strain. To the best of our knowledge, this is the first study to show that dietary probiotics can greatly influence metabolome in fish. Findings of the present study may reveal important implications for maximizing the efficiency of using dietary additives to optimize fish health and growth.

## Introduction

Application of probiotics is one of current approaches for biocontrol and growth promotion in aquaculture (Balcázar et al., 2006). Many different types of bacteria including lactic acid bacteria (LAB) are used as probiotics in fish. In particular, LAB can enhance fish survival by exerting metabolic effects at many levels because LAB can deal with low pH condition and bile acids toxicity presented throughout the gut (Corcoran et al., 2005; Martin et al., 2008). Recent studies have indicated that the use of host-derived probiotics can offer significant advantages in terms of survival and beneficial functions because their physiological activities are at optimum level in the same natural habitats (Lazado et al., 2015; Safari et al., 2016). Improved growth performance and decreased infection rate have been observed in olive flounder (*Paralichthys olivaceus*) fed with a host-derived LAB probiotic strain, *Lactococcus lactis* WFLU12 (Nguyen et al., 2017). Our recent study (Nguyen and Kim, 2018) has demonstrated that strain WFLU12 harbors genes supporting probiotic action based on genome-wide comparison of different *L. lactis* strains.

The growth of animals involves complex processes of protein synthesis and breakdown (Doherty and Whitfield, 2011). The intestine is an important site for the metabolism of dietary nutrients including high levels of protein and amino acids. These nutrients have to traverse intestinal mucosal epithelial cells before entering the bloodstream and body tissues. Metabolome is defined as biochemical compositions of small–molecule metabolites of amino acids, fatty acids, carbohydrates, vitamins, and lipids involved in metabolism. These small-molecule metabolites are required for the maintenance, growth, and normal function of cells (Harrigan and Goodacre, 2003). Metabolome analysis allows us to figure out biochemical reactions being catalyzed by proteins of the proteome which in turn can help us determine biological structure and function of the final phenotype of an organism. In addition, analysis of metabolites produced by intestinal microbiota is an ideal approach to assess health status of the host (Matsumoto et al., 2012). In fish, intestinal bacteria or probiotics possibly produce various kinds of enzymes such as cellulase, amylase, lipase, protease, and chitinase for digestion (Bairagi et al., 2002). They can also regulate the synthesis of specific essential nutrients such as amino acids, fatty acids, and vitamins for fish (Nayak, 2010; Semova et al., 2012). To the best of our knowledge, modes of actions of dietary probiotics used as supplement for fish growth have not been assessed, although large-scale metabolome analysis is rapidly evolving in recent years (Ohashi et al., 2008; Wikoff et al., 2009).

The objective of the present study was to clarify the mode of action involved in the growth promotion effect of probiotics administration in fish. Low-molecular-weight metabolites in samples obtained from healthy fish fed with or without probiotic strain WFLU12 were analyzed and expression levels of metabolites between the two groups were compared to determine changes in the probiotic-fed group. We also attempted to evaluate the benefit of dietary probiotics for host physiology through systemic circulation metabolome analysis. In addition, the whole genome of strain WFLU12 was analyzed to determine any potential genes involved in fish metabolism to increase metabolites.

## Materials and methods

### Ethics approval

Animal experiments performed in this study did not involve any endangered or protected species. All animal experiments were approved by the Animal Ethics Committee of Pukyong National University (Busan, Korea). These experiments were conducted under the guidelines of Animal Ethics Committee Regulations, No. 554 issued by Pukyong National University, Busan, Republic of Korea.

### Animals and probiotic administration

Olive flounder (~165 g and ~26 cm fish^−1^) were purchased from a commercial fish farm in Korea and acclimatized to conditions of aerated and circulated fresh sea water under a 12:12 h light:dark photoperiod for several weeks. After acclimation, fish were randomly divided into a control group and a probiotic-treated group. Control fish were fed a commercial diet (DongA One Corporation, Korea) consisting of crude protein (52%), crude ash (15%), crude fat (10%), phosphorus (2.7%), crude fiber (2%), and calcium (1.2%), while the experimental group of fish were fed a probiotic-supplemented diet (strain WFLU12 at 10^9^ CFU g^−1^ of commercial feed). Strain WFLU12 originally isolated from gastrointestinal tract of wild olive flounder has already been characterized biochemically and genomically in our previous studies (Nguyen et al., 2017; Nguyen and Kim, 2018). Both groups (*n* = 48 for each) were fed at a rate of 3% biomass per day with two daily feedings (9 a.m. and 5 p.m.) for 16 weeks. Weight and length of all the fish in each group were measured every two weeks as described previously (Nguyen et al., 2017). After obtaining growth rate and weight, the ration was adjusted accordingly for the next feeding period. At week 16, nine fish were randomly selected from each group to obtain serum and intestinal mucus samples to obtain three pooled serum and mucus samples for each group. All samples were subjected to analyses of ionic metabolites by CE-TOFMS.

### CE-TOFMS

Metabolomics measurements were performed using an Agilent CE-TOFMS system (Agilent Technologies Inc.). Data were processed with Human Metabolome Technologies Inc. (Tsuruoka, Japan). Values of peaks were normalized to those of methionine sulfone (cationic internal standard) and D-camphor-10-sulfonic acid (anionic internal standard). Detailed information of specimen preparation and CE-TOFMS analysis used in this study is shown in Supplementary Material.

### Ratio of metabolite concentration (probiotics: control group)

In this study, we classified metabolites according to the ratio of values in probiotic-fed group to those in the control group (Pro/Con ratio) as described previously (Matsumoto et al., 2017). We defined threshold Pro/Con ratio to determine whether metabolites were present at higher or lower concentrations in probiotic-fed fish than those in control fish. In this study, the highest Pro/Con ratio for metabolites present at higher concentrations in fish fed with probiotics than those in control fish (the difference was significantly different) was 1.202 (Additional File 3). The lowest Pro/Con ratio for metabolites present at lower concentrations in fish fed with probiotics than those in control fish was 0.830 (Table S1). Therefore, the Pro/Con ratio of metabolites present at higher concentrations in fish fed with probiotics than those in control fish was defined at ≥ 1.20 and the Pro/Con ratio of metabolites present at lower concentrations in fish fed with probiotics than those in control fish was defined at <0.8.

### Prediction of genes of strain WFLU12 involved in increase of metabolites

To find genes of probiotic strain WFLU12 potentially involved in significant increase of metabolites in olive flounder, its whole genome sequence (Accession no. PKRZ00000000) (Nguyen and Kim, 2018) was exploited using KEGG analysis (Moriya et al., 2007).

### Data analysis and statistics

Putative metabolites were assigned from HMT's standard compound library and Known-Unknown peak library based on m/z and MT. If several peaks were assigned to the same candidate, the candidate was given a branch number. Hierarchical cluster analysis (HCA) was performed with PeakStat ver. 3.18 (HMT's in-house software). Principal component analysis (PCA) was performed using SampleStat ver. 3.14 (HMT's in-house software). Differences in relative quantity of individual metabolites between probiotic-fed fish and control fish were evaluated by Welch's *t*-test. The ratio between the two was computed using averaged detection values. Differences in level distribution of whole metabolites between control and probiotic groups were compared using Wilcoxon–Mann–Whitney test. Statistical significance was considered at *p* < 0.05.

## Results

### Viability of probiotic cells in fish gut

Total bacterial counts grown on marine agar for gut mucus retrieved from control and probiotic-fed groups were 5.16 ± 0.72 and 4.79 ± 0.68 log CFU g^−1^ gut mucus, respectively, showing no significant difference between the two groups. Bacterial counts of *L. lactis* (confirmed by PCR) grown on MRS agar recovered from gut mucus of fish in the probiotic-fed group were found to be 4.65 ± 0.61 log CFU g^−1^. However, no LAB was found in gut mucus of fish from the control group.

### Growth measurements

**Figure 1:**
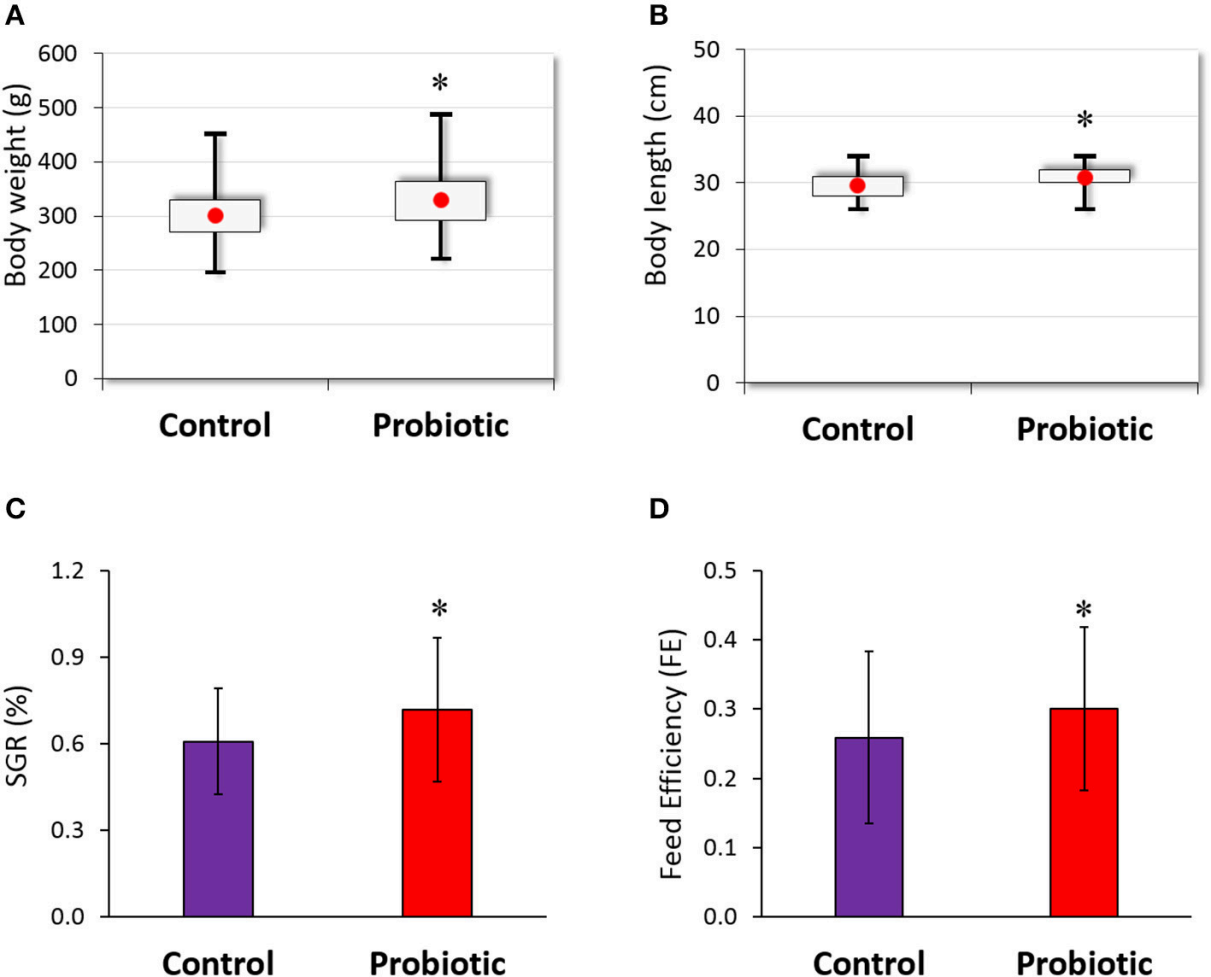
Growth performance of olive flounder (*n* = 48 for each group) fed with or without probiotic *L. lactis* WFLU12 (10^9^ CFU/g of feed) supplemented diets. Weight gain **(A)**, length gain **(B)**, feed efficiency **(C)**, and specific growth rate **(D)** of olive flounder were measured during the experiment period. Each bar represents mean value with standard error (SE). *p < 0.05 compared to the control.

Olive flounder were fed with or without probiotic-containing diet and their growths were monitored for 16 weeks. At the end of the experiment, mean weight and length were 329.98 (g) and 30.77 (cm), respectively, in the probiotic-fed group and 301.06 (g) and 29.60 (cm), respectively, in the control group (Figures 1A,B), showing significant differences between the two groups. Specific growth rate (SGR) and feed efficiency (FE) in the probiotic-fed group were 0.61 ± 0.18 and 0.72 ± 0.25, respectively, which were significantly higher than those (0.26 ± 0.12 and 0.30 ± 0.12, respectively) in the control group (Figures 1C,D).

### Differences in intestinal and serum metabolome between the two dietary groups

CE-TOFMS analyses of compounds present in the prepared pellet (including probiotic-mixed diets-Pellet-P and control diets-Pellet-C) showed 240 compounds, including 148 compounds in cation mode and 92 compounds in anion mode (Additional File 1). Levels of compounds between Pellet-P and Pellet-C were not significantly different (*p* > 0.05 based on Wilcoxon–Mann–Whitney test), indicating that these experimental diets equally distributed compounds to fish in both groups.

**Figure 2:**
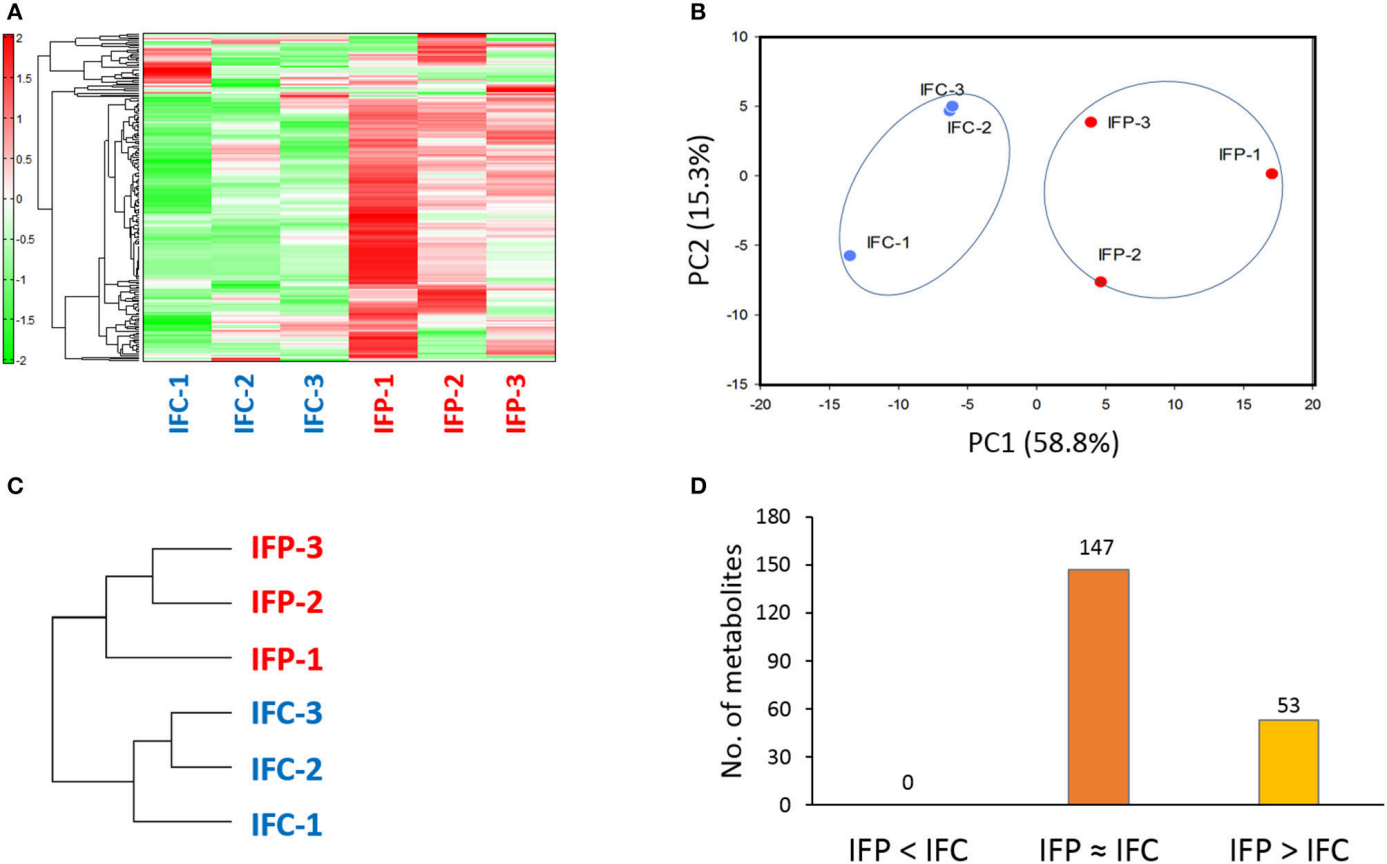
Difference in intestinal metabolome between control fish and probiotic-fed fish. **(A)** Hierarchical clustering showing patterns of intestinal metabolites. Red and green indicate high and low concentrations of metabolites, respectively. **(B)** PCA profiling of data from intestinal metabolome. **(C)** Phylogenetic tree displaying dissimilarity between control and probiotic-fed groups. **(D)** The number of intestinal metabolites in groups IFP > IFC, IFP ≈ IFC, and IFP < IFC.

A total of 200 metabolites (110 cations and 90 anions) were identified from intestinal mucus samples by CE-TOFMS. Patterns of these metabolites from individual samples were subjected to hierarchical clustering and principal component analysis (PCA) as shown in Figure 2. Conspicuously, the intestinal metabolome displayed remarkable difference between the control and probiotic-fed fish groups (Figure 2C), showing that probiotic supplementation significantly modified intestinal metabolome of the host. Of these 200 metabolites, 147 metabolites showed no difference in concentration between the two groups (labeled as IFP ≈ IFC; Additional file 2). However, concentrations of 53 metabolites were significantly (*p* < 0.05) higher or at least 1.5-fold higher (*p* < 0.1) in the probiotic-fed fish group compared to those in the control group (labeled as IFP > IFC) (Figure 2D and Additional file 3).

**Figure 3:**
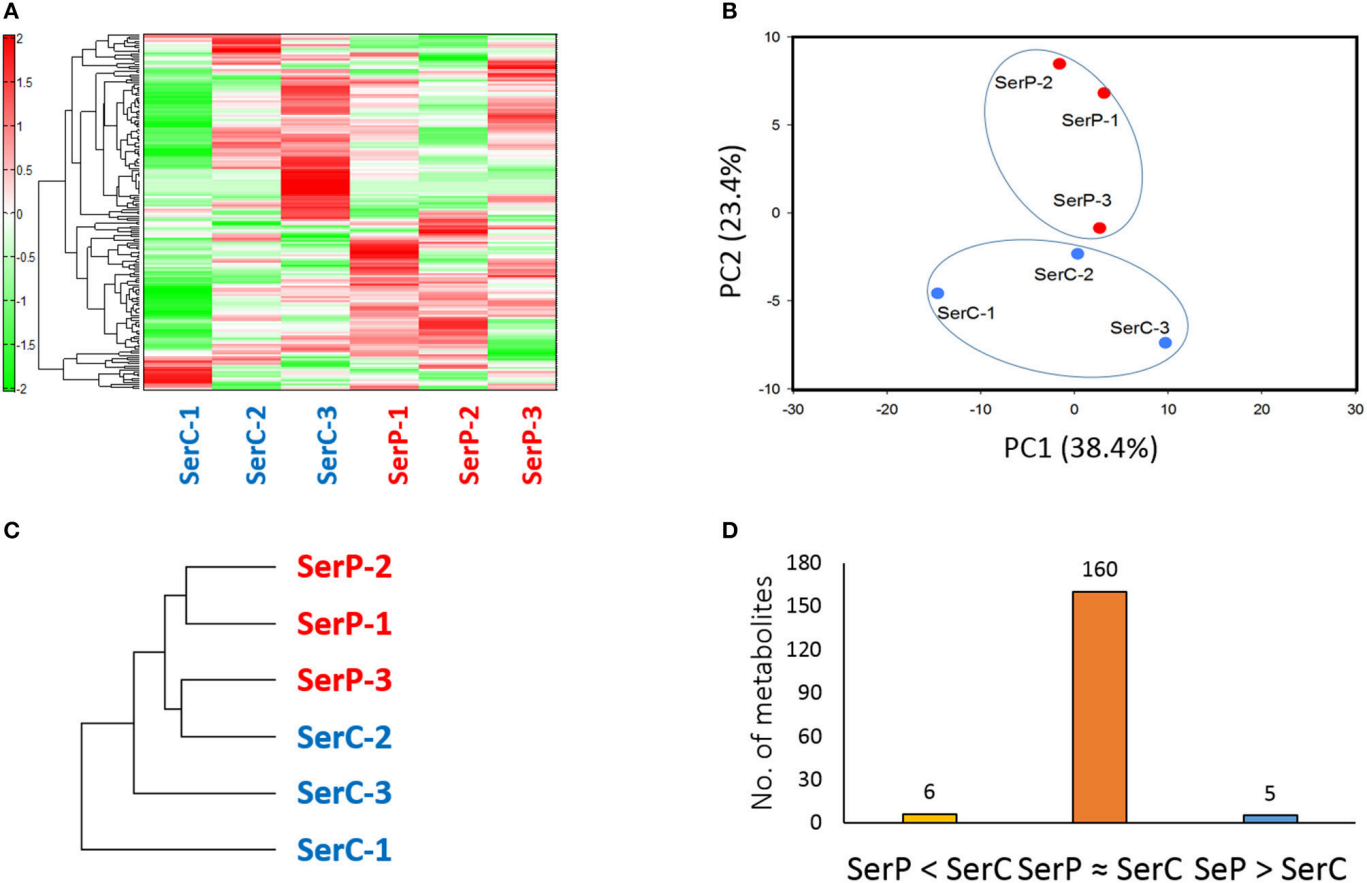
Difference in serum metabolome between control fish and probiotic-fed fish. **(A)** Hierarchical clustering showing patterns of serum metabolites. Red and green indicate high and low concentrations of metabolites, respectively. **(B)** PCA profiling of data from serum metabolome. **(C)** Phylogenetic tree displaying dissimilarity between control and probiotic-fed samples. **(D)** The number of serum metabolites in groups SerP > SerC, SerP ≈ SerC, and SerP < SerC.

A total of 171 metabolites (103 cations and 68 anions) were identified from serum samples from the two fish groups. PCA scores from plot and hierarchical clustering analysis showed that patterns of metabolites in the serum or the degree of clustering did not clearly show distint difference between the two groups (Figure 3). This indicates that probiotic treatment with strain WFLU12 did not significantly change serum metabolic profiles on fish. Mean values of five metabolites (carboxymethyllysine, citrulline, mannosamine, cystathionine, and caffeine) in the probiotic-fed fish group were significantly higher (*p* < 0.05) than those in the control group (denoted as SerP > SerC). However, six metabolites (glucuronic acid-1 or galacturonic acid-1, N,N-Dimethylglycine, prostaglandin F2α, phosphoenolpyruvic acid, homocarnosine, and γ-Glu-2-aminobutyric acid) in the serum were decreased markedly or at least 1.5-fold lower (*p* < 0.1) in the probiotic-fed group than those in the control group (denoted as SerP < SerC) (Table S1). Probiotic-fed fish also showed a tendency to have higher (*p* < 0.1) levels or unique detection of the following metabolites: 5-hydroxylysine, allantoic acid, acetoacetic acid, formiminoglutamic acid, Gln, tyrosine methyl ester, and penicillamine (Additional File 4).

### Classification of metabolites

**Figure 4:**
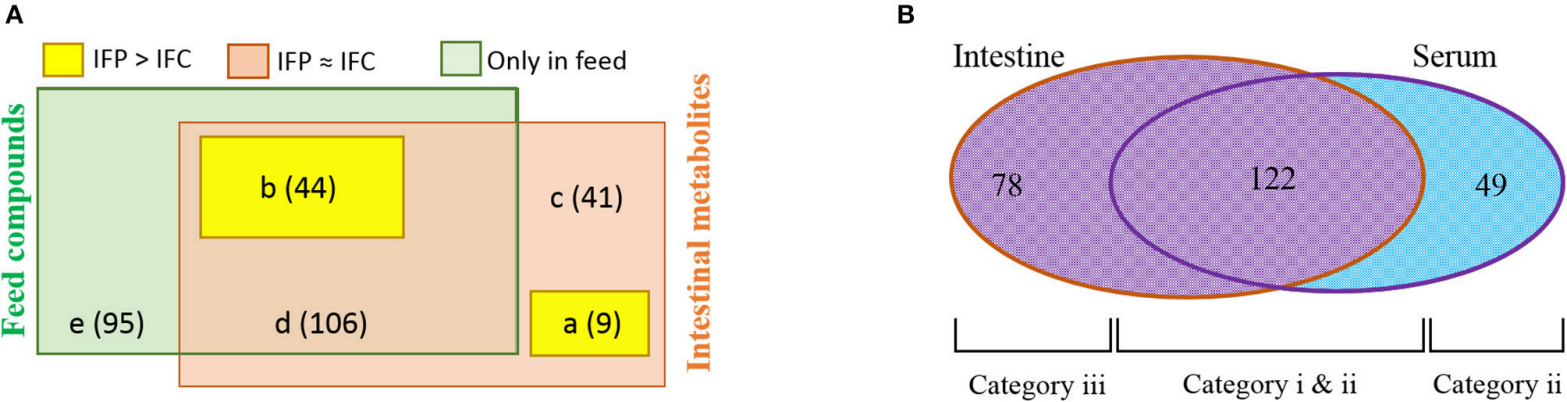
Venn diagram of metabolites and/or compounds noted in the pellet, intestine, and serum metabolome. **(A)** Classification of metabolites and/or compounds detected from fish intestine and pellets (five different groups). **(B)** Classification of metabolites detected from serum and intestine (see additional files and text for details).

To determine whether the probiotic supplement could help fish enhance feed conversion or maintain host physiology, we classified intestinal metabolites by comparing pellet compounds and serum metabolites. We then evaluated the influence of the supplemented probiotic on serum metabolome. Metabolites and/or compounds detected from the intestine and pellet were devived into five different groups: (a) metabolites belonging to group IFP > IFC but not detected from the pellet, (b) metabolites or compounds belonging to group IFP > IFC and detected from the pellet, (c) metabolites belonging to group IFP ≈ IFC but not detected from the pellet, (d) metabolites or compounds belonging to group IFP ≈ IFC and detected from the pellet, and (e) compounds only detected from the pellet (Figure 4A, Additional File 5). Of these metabolite and/or compound groups, this study focuses on groups a and b as they were likely to be invovled in changes in the metabolic processes in fish. Thus, these identified metabolites were classified into 10 functional groups (highlighted in Figure S1 and Additional File 3). In detail, nine metabolites belonging to group a might be produced by host/intestinal microbiota and enriched by the presence of probiotic bacteria in the intestine, while the 44 metabolites belonging to group b were those derived from the pellet and uplifted under the abundant survival of probiotic cells. In particular, amino acid derivatives (10 metabolites), central carbon metabolism intermediates (9 metabolites), and lipid metabolism relatives (8 metabolites) were found to be dominant in group IFP > IFC.

Classification analysis of metabolites detected from fish intestine and serum revealed that 122 (71.3%) of 171 serum metabolites overlapped with intestinal metabolites (Figure 4B), suggesting that they might have been absorbed in the gut and conveyed to the body. A total of 78 metabolites were present only in the intestine profile. They might not have been transported to the body due to filtration by the intestinal wall and/or they are metabolized in internal organs before systemic circulation. The first chemical alteration (metabolization and/or bio-transformation) of many compounds can occur in the colonocyte and the liver (via portal vein) (Matsumoto et al., 2017). A total of 49 (28.7%) metabolites were detected only in the serum, implying that these metabolites might have been derived from metabolism in organs such as kidney, muscle, and liver (Wikoff et al., 2009; Torell et al., 2015). These metabolites detected in this study can be divided into three categories as shown in the Venn diagram (Figure 4B): (1) metabolites (similar pro/con ratios between the intestine and serum) that can be transported from the intestinal lumen to the systemic circulation independent of gateways (category i); (2) metabolites (different pro/con ratios between the intestine and serum) that are influenced by gateways such as the membrane transport system of enterocytes and/or metabolism during transcellular transport (category ii); and (3) metabolites that are not transported to the body or consumed by internal organs(category (iii). Of 122 metabolites (Table S2), 45 (No. 1 to No. 45) belonged to category i. They do not appear to be affected by any gateways. The remaining 77 metabolites (No. 46 to No. 122) belonged to category ii. They might be influenced by gateways.

### Increased metabolites and weight gain

**Figure 5:**
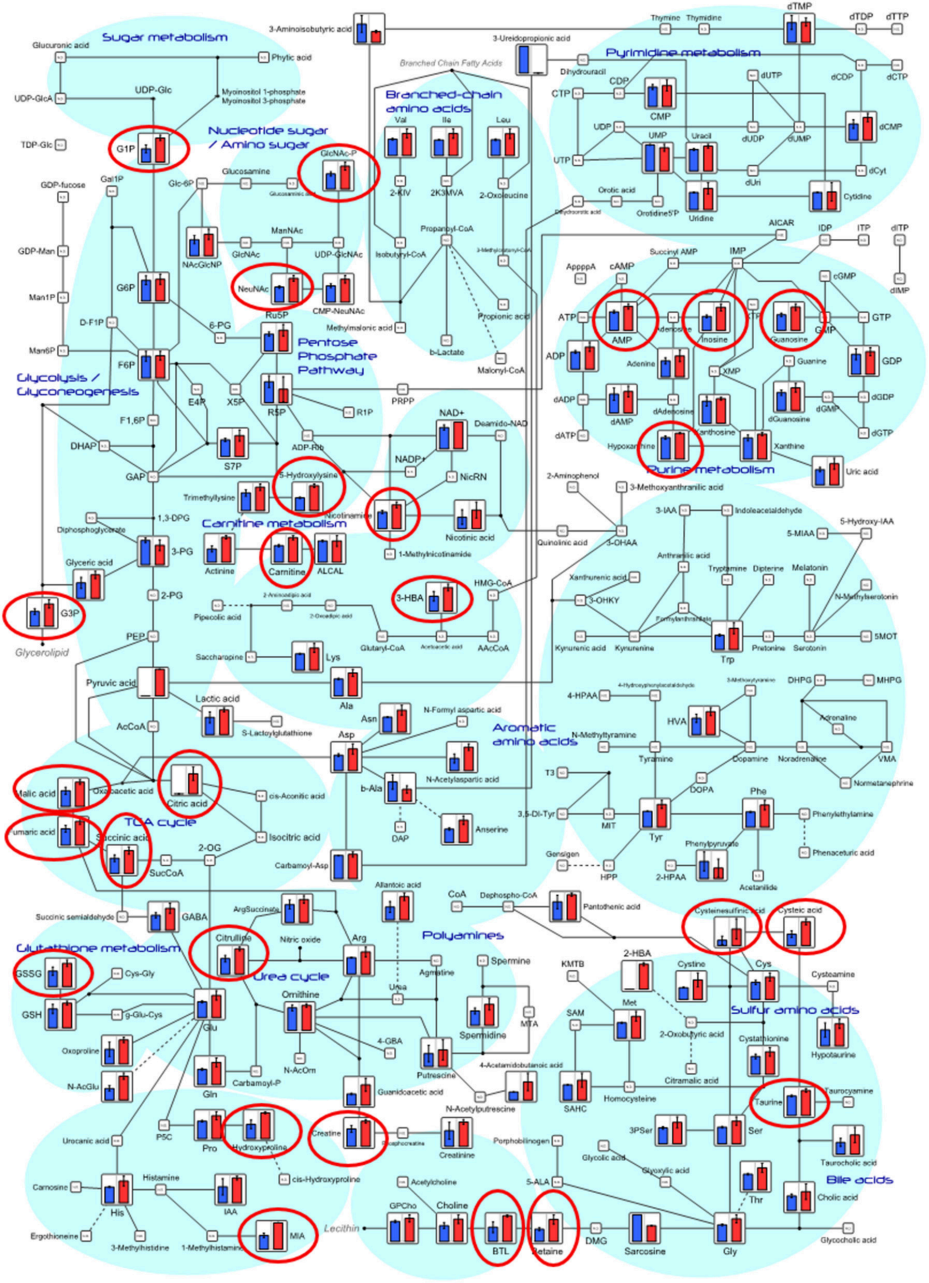
Differences of intestinal metabolites between control fish and probiotic-fed fish on systemic metabolic pathways. Relative quantities of annotated metabolites are represented as bar graphs (blue, control; red, probiotic). Metabolites surrounded by red circles are of higher concentrations in the probiotic group than those in the control group. ND, not detected.

Most metabolites (187 out of 200 in the intestine, and 114 out of 171 metabolites in the serum) detected in probiotic-fed fish had higher concentrations than these in the control (Figure S2). A right-skewed distribution of intestinal metabolite level was particularly indicated in the probiotic-fed group compared to that in the control group (Wilcoxon-Mann-Whitney test: *p* = 0.035 with test variant H1:a_con < b_pro). However, no significant differences in metabolite level was observed for the 171 metabolites detected from fish serum between the two groups. Relative quantities of annotated intestinal metabolites in principal metabolic pathways are represented as bar graphs as shown in Figure 5. Concentrations of metabolites involved in pathways of lipid and amino acid metabolism, polyamine, nucleotide metabolism, and central carbon metabolism were significantly higher in the probiotic-fed group than those in the control group (Table 1). Principal metabolic pathways of serum metabolites are depicted in Figure S3.

**Table 1:** Significantly increased metabolites detected in probiotic-fed fish and their functions.

	**Compound name**	**IFP/IFC**	**SerP/SerC**	**Function**	**References**.
**POLYAMINE AND NUCLEOTIDE METABOLISM**					
Polyamines	N^8^-Acetylspermidine	1.88	ND	A reservoir for spermidine; intestinal maturation	Péres et al., 1997
Nucleotide metabolism	AMP	1.29	ND		
	Guanosine	1.49	1.25		
	Hypoxanthine	1.20	0.94		
	Inosine	1.65	2.34	Enhanced growth performance, survival, feed utilization, immune response, hematological parameters and intestinal morphology	Burrells et al., 2001; Song et al., 2012; Peng et al., 2013
**LIPID AND AMINO ACID METABOLISM**					
Sulfur amino acid metabolism	Taurine	1.28	1.03	Enhance growth; Osmotic pressure regulation; Gut development	Salze and Davis, 2015
	Cysteinesulfinic acid	3.13	ND	Taurine function	
	Cysteic acid	2.07	ND	Taurine function	
	Homocysteic acid	1.97	ND		
Carnitine metabolism	γ-Butyrobetaine	1.90	1.36		
	Carnitine	1.48	0.98	Fat digestibility	Xie et al., 2012
	Butyrylcarnitine	1<	0.74		
	5-Hydroxylysine	1.94	1.31	Collagen function	Li et al., 2009
Choline metabolism	Phosphorylcholine	1.48	1.31	Structure in membrane; neurotransmitter;	Xie et al., 2012
	Betaine	1.79	1.26	Intestinal health	Li et al., 2009; Xie et al., 2012
	Betaine aldehyde_+H_2_O	1.80	ND		
Urea cycle and metabolism of Glu, Gln, His, Pro	1-Methyl-4-imidazoleacetic acid	1.91	1.37		
	Hydroxyproline	1.81	0.99	Enhance growth; Collagen function	Li et al., 2009; Xie et al., 2012
	Citrulline	1.64	2.04	Arginine function	Dhanakoti et al., 1990; Curis et al., 2007
	Homocitrulline	1<	1.04		
	Allantoic acid	1.79	1.20		
	Glutathione (GSSG)_divalent	1.50	1.31	Antioxidant and cell signaling	Li et al., 2009
	N-Acetylglutamic acid	2.06	ND		
Creatine metabolism	Creatine	1.44	1.04	High energy storage; antioxidant; Potential to increase muscle mass	Andersen et al., 2016
**CENTRAL CARBON METABOLISM AND TCA CYCLE**					
TCA	Citric acid	1<	1.17	Improve fish growth, nutrient utilization, and disease resistance	Pan et al., 2004; Sarker et al., 2005
	Fumaric acid	1.45	0.77		
	Malic acid	1.55	0.72		
	Succinic acid	1.51	0.70		
Amino sugar	N-Acetylglucosamine 1-phosphate	1.51	ND		
	N-Acetylneuraminic acid	1.58	1.07		
Nucleotide sugar	Glucose 1-phosphate	1.83	ND		
Glycolysis/Glyconeogenesis	Glycerol 3-phosphate	1.51	0.81		
Keton body	3-Hydroxybutyric acid	1.59	1.10	Storage compound of SCFA; Intestine health;	García Martín et al., 2006; Yagci et al., 2007; den Besten et al., 2013a
**METABOLISM OF COENZYMES**					
Vitamin C metabolism	Ascorbic acid	1.89	ND	Increase the hydroxyproline; improve the growth performance and feed utilization	Fournier et al., 2000; Li et al., 2009; Kim and Kang, 2015
Riboflavin metabolism	FAD_divalent	1.42	ND	Energy metabolism of carbohydrates, fats, and proteins	Shiau and Lin, 2015; LeBlanc et al., 2017
Nicotinamide metabolism	Nicotinamide	1.43	0.95		
Thiamine metabolism	Thiamine	1.69	1.18		
**OTHERS**					
	Carboxymethyllysine	1.53	1.29		
	5-Aminovaleric acid	1.79	ND		
	Methionine sulfoxide	1.69	1.30		
	N-Acetylaspartic acid	1.93	1.03		
	Glucaric acid	1.66	1.25		
	Gluconic acid	1.64	1.12		
	Glucuronic acid-1 Galacturonic acid-1	1.49	0.83		
	Glutaric acid	1.29	0.82		
	Anserine_divalent	1.65	0.91		
	Cysteine glutathione disulfide	1.51	1.04		
	2-(Creatinine-3-yl)propionic acid	1.84	ND		
	2-Hydroxy-4-methylvaleric acid	1.63	1.36		
	4-(β-Acetylaminoethyl)imidazole	2.29	1.59		
	Threonic acid	1.99	1.13		
	XA0004	1.82	1.07		
	XA0033	1.46	1.30		

1<, only detected in probiotic group

*N.D. (Not Detected): Target peak or metabolite was below detection limits*.

### Genes of *L. lactis* WFLU12 involved in increase of metabolites

In the present study, 79 genes of *L. lactis* WFLU12 were found to be involved in 53 increased metabolites in the probiotic-fed group (Table 2). Functions of these genes included energy metabolism and nutrient absorption (e.g., vitamins, TCA cycle intermediates, taurine, and 3-hydroxybutyric acid), gut development (e.g., taurine, citrulline, and polyamines), and regulation of growth and development (e.g., citrulline, hydroxyproline, taurine). A better understanding for functions of genes involved in fish growth is needed because some metabolism pathways are found to be limited or even completely absent in fish due to the lack of host enzymes (for example, limited de novo synthesis of arginine in fish (Li et al., 2009), lack of nucleotides de novo synthesis in intestinal cells (Quan, 1992), and weak capacity of taurine biosynthesis in olive flounder (Wang et al., 2016).

**Table 2:** Genes of *L. lactis* WFLU12 potentially involved in increased metabolites.

**Metabolite**	**Gene description**	**EC number**	**Gene name**	**Locus tag**	**Gene type#**
2-Hydroxy-4-methylvaleric acid	L-2-hydroxyisocaproate dehydrogenase	1.1.1.-		Prokka_01454	Dispensable
3-Hydroxybutyric acid	3-hydroxyisobutyrate dehydrogenase	EC 1.1.1.31	mmsB	Prokka_00965	Dispensable
3-Hydroxybutyric acid	3-ketoacyl-CoA thiolase (EC 2.3.1.16) @ Acetyl-CoA acetyltransferase (EC 2.3.1.9)	EC 2.3.1.16 EC 2.3.1.9	atoB_1	Prokka_02419	Core
3-Hydroxybutyric acid	3-ketoacyl-CoA thiolase (EC 2.3.1.16) @ Acetyl-CoA acetyltransferase (EC 2.3.1.9)	EC 2.3.1.16 EC 2.3.1.9	atoB_2	Prokka_00418	Dispensable
AMP	Adenine phosphoribosyltransferase	EC 2.4.2.7	apt	Prokka_01595	Core
AMP	Adenylosuccinate lyase	EC 4.3.2.2	purB	Prokka_00224	Core
AMP, thiamine	Adenylate kinase	EC 2.7.4.3	adk	Prokka_00825	Core
Ascorbic acid	PTS system, ascorbate-specific IIC component	EC:2.7.1.194	ulaC	Prokka_00208	Dispensable
Ascorbic acid	PTS system, ascorbate-specific IIA component		ulaA	Prokka_00206	Dispensable
Carboxymethyllysine	N5-(carboxyethyl)ornithine synthase	EC:1.5.1.24	ceo	Prokka_01676	Core
Citric acid	Aconitate hydratase	EC 4.2.1.3	acnA	Prokka_01615	Core
Citric acid	Citrate synthase (si)	EC 2.3.3.1	gltA	Prokka_01614	Core
Citrulline	Acetylglutamate kinase	EC:2.7.2.8	argB	Prokka_01842	Core
Citrulline	Acetylornithine deacetylase	EC:3.5.1.16	argE	Prokka_01527	Core
Citrulline	Acetylornithine/N-succinyldiaminopimelate aminotransferase	EC:2.6.1.11 2.6.1.17	argD	Prokka_01841	Dispensable
Citrulline	Alanine-synthesizing transaminase	EC:2.6.1.66 2.6.1.2	alaA	Prokka_01171	Core
Citrulline	Arginine deiminase	EC:3.5.3.6	arcA	Prokka_00791	Core
Citrulline	Argininosuccinate lyase	EC:4.3.2.1	argH	Prokka_01135	Core
Citrulline	Carbamate kinase	EC:2.7.2.2	arcC	Prokka_00276	Core
Citrulline	Carbamate kinase	EC:2.7.2.3	arcC	Prokka_00787	Core
Citrulline	Carbamate kinase	EC:2.7.2.4	arcC	Prokka_00788	Core
Citrulline	Carbamoyl-phosphate synthase large subunit	EC:6.3.5.5	carB	Prokka_00118	Core
Citrulline	Carbamoyl-phosphate synthase small subunit	EC:6.3.5.5	carA	Prokka_00181	Core
Citrulline	Glutamate N-acetyltransferase / amino-acid N-acetyltransferase	EC:2.3.1.35 EC:2.3.1.1	argJ	Prokka_01840	Dispensable
Citrulline	Glutamine synthetase	EC:6.3.1.2	glnA	Prokka_00951	Dispensable
Citrulline	N-acetyl-gamma-glutamyl-phosphate reductase	EC:1.2.1.38	argC	Prokka_01839	Core
Citrulline	Argininosuccinate synthase	EC:6.3.4.5	argG	Prokka_01134	Core
Citrulline	Ornithine carbamoyltransferase	EC:2.1.3.3	argF	Prokka_00790	Core
Citrulline	Ornithine carbamoyltransferase	EC:2.1.3.4	argF	Prokka_01843	Core
Cysteic acid	L-serine dehydratase	EC:4.3.1.17	sdaA	Prokka_01865	Core
Cysteic acid	L-serine dehydratase	EC:4.3.1.18	sdaB	Prokka_01866	Core
Cysteic acid	Phosphoadenosine phosphosulfate reductase	EC:1.8.4.8	cysH	Prokka_02147	Dispensable
Cysteic acid	Phosphoadenosine phosphosulfate reductase	EC:1.8.4.8	cysH	Prokka_02536	Dispensable
Cysteic acid	Sulfite exporter TauE/SafE			Prokka_00969	Dispensable
Cysteic acid	Aspartate aminotransferase	EC 2.6.1.1	aspB	Prokka_00474	Core
Cysteine desulfurase	Cysteine desulfurase	EC:2.8.1.7	iscS	Prokka_00504	Core
Cysteine desulfurase	Cysteine desulfurase	EC:2.8.1.8	iscS	Prokka_01482	Core
FAD_divalent	5-amino-6-(5-phospho-D-ribitylamino)uracil phosphatase	EC:3.1.3.104	ycsE	Prokka_00365	Core
FAD_divalent	6,7-dimethyl-8-ribityllumazine synthase	EC:2.5.1.78	ribH	Prokka_02055	Core
FAD_divalent	Diaminohydroxyphosphoribosylaminopyrimidine deaminase / 5-amino-6-(5-phosphoribosylamino)uracil reductase	EC:3.5.4.26 EC:1.1.1.193	ribD	Prokka_02052	Core
FAD_divalent	FMN hydrolase / 5-amino-6-(5-phospho-D-ribitylamino)uracil phosphatase	EC:3.1.3.102 EC:3.1.3.104	ybjI	Prokka_00055	Dispensable
FAD_divalent	Riboflavin kinase / FMN adenylyltransferase	EC:2.7.1.26 EC:2.7.7.2	ribF	Prokka_02136	Core
FAD_divalent	Riboflavin synthase	EC:2.5.1.9	ribE	Prokka_02053	Core
FAD_divalent	3,4-dihydroxy 2-butanone 4-phosphate synthase / GTP cyclohydrolase II	EC:4.1.99.12 EC:3.5.4.25	ribBA	Prokka_02054	Core
Fumaric acid	Fumarate reductase, flavoprotein subunit precursor	EC 1.3.99.1		Prokka_01381	Core
Glucose 1-phosphate	Phosphoglucomutase	EC 5.4.2.2	pgmB	Prokka_01441	Core
Glutathione (GSSG)_divalent	Glutathione peroxidase	EC 1.11.1.9	gpx	Prokka_00119	Core
Glutathione (GSSG)_divalent	Glutathione reductase	EC 1.8.1.7	gor	Prokka_01883	Core
Glycerol 3-phosphate	2,3-bisphosphoglycerate-dependent phosphoglycerate mutase	EC:5.4.2.11	PGAM	Prokka_00347	Core
Glycerol 3-phosphate	2,3-bisphosphoglycerate-dependent phosphoglycerate mutase	EC:5.4.2.12	PGAM	Prokka_01350	Core
Glycerol 3-phosphate	Phosphoglycerate kinase	EC 2.7.2.3	pgk	Prokka_01250	Core
Guanosine	Purine nucleoside phosphorylase	EC 2.4.2.1	deoD	Prokka_01998	Core
Hydroxyproline	Pyrroline-5-carboxylate reductase	EC:1.5.1.2	proC	Prokka_00530	Core
Hydroxyproline	Pyrroline-5-carboxylate reductase	EC:1.5.1.3	proC	Prokka_02427	Dispensable
Inosine	Adenosine deaminase	EC:3.5.4.4	add	Prokka_01301	Dispensable
Inosine	Cytidine deaminase	EC:3.5.4.5	cdd	Prokka_02546	Core
Malic acid	malate dehydrogenase	EC 1.1.1.38	maeA	Prokka_01968	Core
Methionine sulfoxide	Peptide-methionine (R)-S-oxide reductase	EC:1.8.4.12	msrB	Prokka_01199	Core
Methionine sulfoxide	Peptide-methionine (S)-S-oxide reductase	EC:1.8.4.11	msrA	Prokka_00761	Core
Methionine sulfoxide	Peptide-methionine (S)-S-oxide reductase	EC:1.8.4.12	msrA	Prokka_02442	Dispensable
N^8^-Acetylspermidine	diamine N-acetyltransferase	EC:2.3.1.57	speG	Prokka_00195	Dispensable
N-Acetylglucosamine 1-phosphate	N-acetylglucosamine-1-phosphate uridyltransferase / Glucosamine-1-phosphate N-acetyltransferase	EC 2.7.7.23 /EC 2.3.1.157	glmU	Prokka_00529	Core
N-Acetylglutamic acid	N-acetylglutamate synthase	EC 2.3.1.1	Nags	Prokka_01559	Core
N-Acetylneuraminic acid	Phosphoglucosamine mutase	EC 5.4.2.10	glmM	Prokka_01436	Core
Nicotinamide	NAD+ kinase	EC 2.7.1.23	ppnK	Prokka_01377	Core
Nicotinamide	NAD+ synthetase	EC 6.3.1.5	nadE	Prokka_02113	Dispensable
Nicotinamide	Nicotinate-nucleotide adenylyltransferase	EC 2.7.7.18	nadD	Prokka_02109	Core
Nicotinamide	N-Ribosylnicotinamide phosphorylase	EC 2.4.2.1	deoD	Prokka_01998	Core
Nicotinamide	Nicotinate phosphoribosyltransferase	EC 6.3.4.21	pncB	Prokka_02111	Core
Taurine	Glutamate decarboxylase	EC 4.1.1.15	gadB	Prokka_00031	Dispensable
Thiamine	1-deoxy-D-xylulose-5-phosphate synthase	EC:2.2.1.7	dxs	Prokka_00257	Dispensable
Thiamine	1-deoxy-D-xylulose-5-phosphate synthase	EC:2.2.1.8	dxs	Prokka_02507	Dispensable
Thiamine	Cysteine desulfurase / selenocysteine lyase	EC:2.8.1.7	sufS	Prokka_00426	Core
Thiamine	Hydroxyethylthiazole kinase	EC:2.7.1.50	thiM	Prokka_02295	Core
Thiamine	Ribosome biogenesis GTPase / thiamine phosphate phosphatase	EC:3.6.1.- 3.1.3.100	rsgA	Prokka_00641	Core
Thiamine	Thiaminase (transcriptional activator TenA)	EC:3.5.99.2	tenA	Prokka_00414	Core
Thiamine	Thiamine-phosphate pyrophosphorylase	EC:2.5.1.3	thiE	Prokka_02293	Core
Thiamine	tRNA uracil 4-sulfurtransferase	EC:2.8.1.4	thiI	Prokka_01389	Core
Thiamine	Hydroxymethylpyrimidine/phosphomethylpyrimidine kinase	EC:2.7.1.49 EC:2.7.4.7	thiD	Prokka_02294	Core

*# Genes were identified based on pan-genome analysis using genome sequence of 20 L. lactis strains including the strain WFLU12 (Nguyen and Kim, 2018). The 19 strains include bacteria isolated from dairy product, UL8, C10, UC063, 275, 229, Il1403, 184, KLDS 4.0325, and SO; plant, AI-06, KF147, and NCDO 2118; meat, UC08 and UC11; drain water, IO-01; fermented food, A12; and human, CV56*.

## Discussion

In this study, concentrations of metabolites measured in the probiotic-fed group were higher overall than those in the control group. In particular, concentrations of 53 and 5 metabolites were significantly higher in intestine and serum samples, respectively, of the probiotic-fed group than those in the control group. However, the number of significantly increased metabolites in the intestine differed somewhat from that in the serum of the probiotic-fed group. Such discrepancy might be due to several gateways from the gut lumen to systemic circulation in the blood. In this study, 45 (category i) metabolites were transported to the blood without being influenced by gateways while 77 (category ii) metabolites influenced by gateways were transported to the blood. Indeed, the types of metabolites that can and cannot be transported to the blood from the lumen remains unclear. A previous study (Matsumoto et al., 2017) showed that 11 basic amino acids including Phe, Lys, Ser, Gly, His, Thr, Arg, Pro, and branched chain amino acids (BCAAs) are transported independently from the colon to systemic circulation. In this study, however, most of the basic amino acids (18; see Table S2) were found to be filtrated and/or metabolized. This concurs with a previous study (Wu, 1998) showing that dietary amino acids can be catabolized by the small intestinal mucosa in the first pass. The intestine can modulate the availability of amino acids to extra-intestinal tissues. For example, the differences between the Pro/Con ratio of Arg (No. 74) and citrulline (No. 44) were observed in the intestinal mucus and serum samples in the present study. Citrulline is mainly synthesized from the conversion of glutamine in the enterocyte. Other amino acids such as arginine, proline, and ornithine can be converted into citrulline, and then released into the blood stream as an important source of endogenous arginine (Curis et al., 2007). Therefore, the higher Pro/Con ratio of citrulline in the serum than that in the intestine might be due to the metabolism of the amino acids by enterocytes. Moreover, the gastrointestinal tissue has been demonstrated to possess enzymes needed to metabolize methionine to cysteine (Finkelstein, 2000) or perform methionine transmethylation to release homocysteine and cystathionine into the circulation (Townsend et al., 2004). In this study, cysteine was only detected in intestinal mucus, and not in the pellet or serum (Additional File 2), indicating that methionine can be converted to cysteine by gut microbiota and enterocytes, and/or cysteine might be metabolized in the liver before systemic circulation. Cysteine might be extensively metabolized by intestinal enterocytes and the liver via cysteine dioxygenase to form cysteinesulfinate (Malmezat et al., 2000), a precursor of taurine. Higher Pro/Con ratio of cystathionine (No. 43) in serum metabolome might indicate transmethylation of methionine in the intestine. Cystathionine in the serum can be an important source of cysteine and glutathione synthesis in the body (Shoveller et al., 2005). Therefore, many metabolites might be influenced by intestinal metabolism. Pro/Con ratios of metabolites obtained in the present study are important for understanding the bioavailability of metabolites in fish.

It has been demonstrated that host circulating metabolites are linked to gut microbial species in human. They can cause body weight change by altering host energy harvest and storage (e.g., Wahl et al., 2015; Liu et al., 2017). Many studies have shown that the dietary supplementation of probiotics in farmed animals including fish can promote their growth without increasing fat mass (Khaksefidi and Rahimi, 2005; Šabatková et al., 2008). These observations might be due to improved gut function and resistance to infection rather than metabolic imbalance that causes overweight. Rogge (2009) has indicated that overweight is associated with decreased fatty acid β-oxidation. Therefore, overweight individuals are more dependent on the glycolytic pathway for ATP production, resulting in an increase in pyruvate production. The impact of gut microbiome on serum metabolome alterations has also been reported, showing that serum concentrations of many targeted metabolomics profiling of amino acids such as citrulline, cystine, lysine, and leucine are considerably higher in overweight individuals than those in lean controls (Liu et al., 2017). In our study, serum concentrations of citrulline, cystathionine (as an intermediate of sulfur amino acid cysteine), 2-hydroxy-4-methylvaleric acid (leucine derivative), and 5-hydroxylysine (an intermediate of lysine; 1.3-fold change; *p* < 0.1) were considerably higher in the probiotic-fed group of fish than those in the control group (Table S1, Additional File 4). The concentration of allantoic acid in the serum of the probiotic-fed group was also significantly higher compared to that in the control group (1.2-fold change; *p* = 0.05; Table 1 and Additional File 4). In most fish, the formed allantoin is further degraded to urea and glyoxalate via allantoic acid by allantoicase and allantoinase (Alvarez-Lario and Macarrón-Vivente, 2011). Since allantoin can be used for identifying serum uric acid level and predicting weight gain in humans (Masuo et al., 2003), increased level of this compound in the serum could be a sign of weight gain in fish.

Metabolites including amino acids are not only major fuels for intestinal mucosa, but also essential precursors for intestinal synthesis of glutathione, nitric oxide, polyamines, purine, and pyrimidine nucleotides as well as amino acids (e.g., citrulline, cysteine) which in turn will maintain intestinal mucosal mass and integrity (Wu, 1998). In this study, concentrations of citrulline in both the intestine and serum of probiotic-fed fish were significantly higher than those in the control. In general, arginine has a very weak gastrointestinal uptake rate. However, citrulline can be easily taken up in the gut and then converted to arginine in the kidney (Dhanakoti et al., 1990; Curis et al., 2007). The gut is known as the primary organ responsible for most circulating citrulline synthesis from glutamine (with ornithine carbamoyltransferase) (Wu, 1998; Bolotin et al., 2001). However, de novo synthesis of arginine in fish is limited or completely absent due to the lack of pyrroline-5-carboxylate (P5C) synthase in fish (Li et al., 2009). P5C is the key enzyme involved in the synthesis of proline and ornithine from glutamine/glutamate. These intermediates are then further metabolized to citrulline and arginine (Li et al., 2009). Therefore, fish have particularly high requirements for dietary arginine as a nutritionally indispensable and essential amino acid for growth. Kao et al. (2016) have shown that the unique gut microbiota might contribute to altered arginine and citrulline metabolism. It is well known that arginine is converted to citrulline through arginine deiminase (ADI) pathway (Noens et al., 2015). Similar to other *L. lactis* strains such as strain IL-1403 (Bolotin et al., 2001) and ATCC 7962 (Kim et al., 2007), strain WFLU12 harbors biosynthetic genes involved in the ADI pathway (Table 2). It has been demonstrated that the ADI pathway of *L. lactis* is a biocatalyst for citrulline production with a very high specific activity (140.3 U/mg) (Kim et al., 2007; Song et al., 2015).

Concentrations of polyamine (N8-acetylspermidine) and creatine in probiotics-fed fish were significantly higher than those in the control group (Table 1). This might be due to sufficient production of citrulline. Arginine derived from a precursor of citrulline can be catabolized to intermediates such as ornithine and guanidinoacetic acid (Figure S4), yielding polyamine and creatine as previously described (Morris, 2009; Andersen et al., 2016). Creatine is used as a storage molecule for energy in the form of phosphocreatine in the muscle. It has potential to increase muscle mass (Andersen et al., 2016). Creatine metabolism plays an essential role in epithelial cell barrier function and intestinal homeostasis in mice (Turer et al., 2017). Polyamines and their acetylated derivatives are essential in regulating and renewing intestinal mucosa (Gao et al., 2013; Ramani et al., 2014). Péres et al. (1997) have demonstrated that dietary polyamine spermine can increase digestive enzyme activity and promote intestinal maturation in sea bass larvae (*Dicentrarchus labrax*). Taken together, these results suggest that increased turnover of polyamine and creatine might be related to the increase of citrulline in probiotic-fed fish, implying its potential effects on energy metabolism and maintenance of robust gut health (Andersen et al., 2013) as well as symbiotic probiotics–host metabolic interactions.

It has been demonstrated that dietary supplementation with nucleotides (e.g., inosine, guanosine) can enhance the growth performance of many fish species, including olive flounder (Burrells et al., 2001; Song et al., 2012). Nucleotides might be very important for improving intestinal morphology (Cheng et al., 2011; Peng et al., 2013) because intestinal cells lack the ability to de novo synthesize nucleotides and must depend on exogenous supply (Quan, 1992). In the present study, purine derivatives including inosine, hypoxanthine, AMP, and guanosine were increased in the probiotic-fed group (Table 1, Figure S5), indicating that dietary supplementation of strain WFLU12 could induce de novo synthesis of purines in the intestine to meet the requirement for growth. Strain WFLU12 possesses gene *add* encoding adenosine deaminase (EC 3.5.4.4, Table 2). This gene can convert adenosine to inosine for utilizing general purine sources (Martinussen et al., 2003; Kilstrup et al., 2005).

In this study, concentrations of taurine and its precursors such as cysteic acid, homocysteic acid, and cysteinesulfinic acid were found to be significantly increased in the intestine of the probiotic-fed group compared to those in the control (Table 1; Figure S6). Taurine is a sulfonic acid found in high concentrations in animal tissues, including olive flounder (Sakaguchi and Murata, 1988; Jang et al., 2011). In recent years, several studies have demonstrated the essentiality (i.e., improved growth and survival) of dietary taurine for many commercially relevant species, especially marine teleosts including olive flounder (Park et al., 2002; Kim et al., 2003, 2008; Salze and Davis, 2015). Previous studies (Park et al., 2002; Wang et al., 2016) have demonstrated that olive flounder has a very weak taurine biosynthetic capability due to a mutation in enzyme cysteic acid decarboxylase (CAD). In the present study, KEGG analysis of *L. lactis* genome showed that it possessed genes encoding enzymes that might be involved in the biosynthesis of cysteic acid (Table 2) via serine/sulfate pathway (Machlin et al., 1955; Tevatia et al., 2015). Gene *gad*B (EC 4.1.1.15) encoding glutamate decarboxylase beta can potentially decarboxylate cysteic acid to yield taurine. In this study, analysis of molecular dynamics simulations and homology modeling for GAD protein of *L. lactis* WFLU12 and its substrates showed that both CSA and CA simulation shared binding sites of pyridoxal-5′-phosphate in GAD of the *L. lactis* structural model (Figure S7, Supplementary Material). Thus, GAD of *L. lactis* might be potentially involved in the bio-conversion of taurine and dietary supplementation of *L. lactis* might have contributed to the completion of the biosynthetic pathway of taurine in fish intestine.

It is worth noting that concentrations the 3-hydroxybutyric acid (3HBA) and 2-hydroxy-4-methylvaleric acid (HICA) values were significantly higher in the probiotic-fed group than those in the control group (Table 1). 3HBA is a storage compound of short chain fatty acids (SCFA) acetate (García Martín et al., 2006; Yagci et al., 2007) derived from fermentation of dietary fibers by intestinal microbiota (den Besten et al., 2013a,b). Amino acid derivative HICA is a protein-fermentation product of bacteria such as lactobacilli (Smit et al., 2004). The increase of 3HBA was correlated with increased acetoacetic acid (also diacetate) in the serum (Additional File 4), indicating that these ketone bodies might be readily reconverted to acetyl-CoA to produce energy (Laffel, 1999). Hence, metabolic shift of SCFA in the gut of fish can beneficially aid in nutrition source and ATP production for cells lining the intestine (den Besten et al., 2013b; Jost et al., 2014). It has been revealed that HICA can increase weight and muscle mass in chickens and rats (Boebel and Baker, 1982). In the present study, genes encoding enzyme 3-hydroxyisobutyrate dehydrogenase for conversion of 3HBA to acetoacetic acid and L-2-hydroxyisocaproate dehydrogenase (HicDH) for HICA production (de Cadiñanos de et al., 2013) were found in our probiotic genome (Table 2). However, functions of these metabolites in fish are not yet fully understood. Thus, further study is needed to clarify their functions in fish.

Concentrations of several metabolites involved in the TCA cycle were increased significantly in the intestine of fish fed with probiotics (Figure S8). TCA cycle intermediates are known to be able to improve growth, nutrients absorption, and bioavailability of minerals in several fish species (e.g., Pan et al., 2004; Sarker et al., 2005). In addition, increased carnitine, a key metabolite involved in fatty acid catabolism by β-oxidation after esterification to release stored energy from fatty acid (Xie et al., 2012) in the intestine of probiotic-fed group (Table 1), might be related to improvement of fat digestibility, thus increasing citric acid production through AcCoA intermediate (Figure S7). Digesting and absorbing dietary nutrients are critical physiological functions of the intestine necessary for maximal growth of the organism. In this context, the intestine has a high rate of energy expenditure because digestion and absorption processes are critically dependent on energy (Yang et al., 2016). Results of the present study suggest that energy generated from the citric acid cycle in the intestine might benefit fish digestion and absorption of nutrients in the probiotic-fed group, thus contributing to significant growth improvement.

Vitamins are important organic compounds that are essential for optimal growth and the health of animals. In general, vitamins are not synthesized by fish. They need to be supplied in the diet (Craig and Helfrich, 2009). It is well known that intestinal bacteria as well as lactic acid bacteria can synthesize and supply various vitamins (LeBlanc et al., 2013). In the present study, we found that fish in the probiotic-fed group showed increased levels of vitamin C (Table 1), known to be related to improvement of growth performance and feed utilization in different fish species (Fournier et al., 2000; Kim and Kang, 2015). In this study, increased level of hydroxyproline in the probiotic-fed group might be related to post-translational hydroxylation of proline by vitamin C-dependent prolyl hydroxylase (Li et al., 2009). Members of vitamin B complex are also needed in fish diet as they play major roles in growth, physiology, and cellular metabolism (Shiau and Lin, 2015). In this study, levels of co-enzymes such as riboflavin, thiamine, and nicotinamide were increased in fish fed with probiotics compared to those in the control group (Table 1), indicating that they might play an important role in energy metabolism of carbohydrates, fats, and proteins (LeBlanc et al., 2017). Similar to other *L. lactis* strains (Burgess et al., 2004; Shimizu-Kadota et al., 2013), our strain possesses various genes involved in the bioconversion of vitamins (Table 2). These features of strain WFLU12 might confer fish metabolic versatility of converting materials for energy in fish intestine.

Previous studies (e.g., Drissi et al., 2014) on weight gain-associated *Lactobacillus* spp. have shown that these *Lactobacillus* encode ubiquitous enzymes with key roles in β-oxidation pathway of fatty acid degradation (Thiolase I; EC 2.3.1.16) and various biosynthetic pathways (Thiolase II; EC 2.3.1.9) such as poly β-hydroxybutyric acid synthesis and steroid biogenesis (Haapalainen et al., 2006). Thus, genomes of weight gain-associated *Lactobacillus* might be able to mobilize energy and carbon stored in fatty acids through β-oxidation. In agreement with this, our strain harbored both thiolase I and II (Table 2). These might be responsible for the increase of poly β-hydroxybutyric acid and β-oxidation pathway (level of carnitine in Table 1). This is another evidence supporting that our probiotic strain could increase different kinds of metabolites as observed in this study.

## Conclusion

Dietary supplementation with *L. lactis* WFLU12 greatly influenced metabolism in fish. Several metabolites including citrulline, TCA cycle intermediates, SCFA, vitamins, and taurine are likely to be linked to growth promotion in fish. In particular, our probiotic strain has a number of genes encoding diverse enzymes to help many important metabolic pathways such as urea cycle relating metabolism, taurine biosynthesis, and de novo synthesis of nucleotides in the intestine. In the field of gastrointestinal physiology, our pioneering study demonstrates that intestinal catabolism plays a very important role in modulating metabolite availability to extra-intestinal tissues. Our data also provide novel insights into the metabolomic mode of action of probiotics involved in fish health and growth.

## Author contributions

TN designed the experiments, performed data processing and analysis, and wrote the paper. W-KC, AK, NK, HR, and YL performed experiments. MY performed experiments and analyzed the data. SK and C-IP analyzed the data. D-HK conceived and designed the experiments, and wrote the paper. All authors edited the manuscript and approved the final version.

### Conflict of interest statement

The authors declare that the research was conducted in the absence of any commercial or financial relationships that could be construed as a potential conflict of interest.

## Supplementary material

Figure S1 Classification of intestinal metabolites in four different groups displayed in Figure 4A. Identified metabolites were classified into 10 functional groups (see main text for details).

Figure S2 Ratio of metabolite concentration in probiotic-fed vs. control. **(A)** Intestinal metabolites. **(B)** Serum metabolites. The ratio is computed by using averaged detection values.

Figure S3 Differences of serum metabolites between control fish and probiotic-fed fish on systemic metabolic pathways. Relative quantities of annotated metabolites are presented as bar graphs (green, control; orange, probiotic). Metabolites surrounded by red circles are of higher concentrations in the probiotic group than those in the control group. ND, not detected.

Figure S4 Differences of intestinal metabolites between control fish and probiotic-fed fish on urea cycle related metabolism pathways. Relative areas of annotated metabolites are presented as bar graphs (blue, control; red, probiotic). Metabolites surrounded by red circles are of higher concentrations in the probiotic group than those in the control group. ND, not detected.

Figure S5 Differences of intestinal metabolites between control and probiotic fish on purine metabolism pathways. Relative quantities of annotated metabolites are presented as bar graphs (blue, control; red, probiotic). Metabolites surrounded by red circles are of higher concentrations in the probiotic group than those in the control group. ND, not detected.

Figure S6 Differences of intestinal metabolites between control fish and probiotic-fed fish on lipid and amino acid metabolism pathways. Relative areas of annotated metabolites are presented as bar graphs (blue, control; red, probiotic). Metabolites surrounded by red circles are of higher concentrations in the probiotic group than those in the control group. ND, not detected.

Figure S7 Binding sites of pyridoxal-5′-phosphate (PLP) and substrates in *L. lactis* GAD. The overall dimeric structure of LcGAD is indicated by ribbons and colored by blue and red for each chain (A). PLP and substrates (B: L-Glu, C: CSA, D: CA) are presented as thick sticks and residues forming the binding site are presented as thin sticks. Carbon, nitrogen, oxygen, phosphorus, and sulfur atoms are colored by cyan, blue, red, gold, and yellow, respectively.

Figure S8 Differences of intestinal metabolites between control fish and probiotic-fed fish on the central carbon metabolism pathways. Relative areas of annotated metabolites are presented as bar graphs (blue, control; red, probiotic). Metabolites surrounded by red circles are of higher concentrations in the probiotic group than those in the control group. ND, not detected.

Table S1 List of metabolites significantly changed in the serum. 1<, only detected in probiotic group. N.D. (Not Detected): Target peak or metabolite was below detection limits.

Table S2 Pro/Con ratio and possible influence of gateways on each metabolite detected in the intestine and serum.

Table S3 Binding sites of PLP and substrates in three simulation systems.

Additional File 1 Compounds detected in the pellet mixed with probiotics (Pellet-P) and control pellet (Pellet-C).

Additional File 2 List of 147 metabolites in group IFP ≈ IFC.

Additional File 3 List of 53 intestinal metabolites increased significantly in probiotic-fed fish (group IFP > IFC).

Additional File 4 List of 160 metabolites showing no significant change in the serum.

Additional File 5 Additional information on metabolite classification (Figure 4).
