# Supplementary material for: Dietary Probiotic Effect of Lactococcus lactis WFLU12 on Low-Molecular-Weight Metabolites and Growth of Olive Flounder (Paralichythys olivaceus)
Source: Front Microbiol. 2018 Sep 5;9:2059. doi: 10.3389/fmicb.2018.02059 (PMC6134039; doi:10.3389/fmicb.2018.02059)
Supplement: Figure S1 — Classification of intestinal metabolites in four different groups displayed in Figure 4A. Identified metabolites were classified into 10 functional groups (see main text for details). [file Presentation_1.PPTX]

## Slide 1
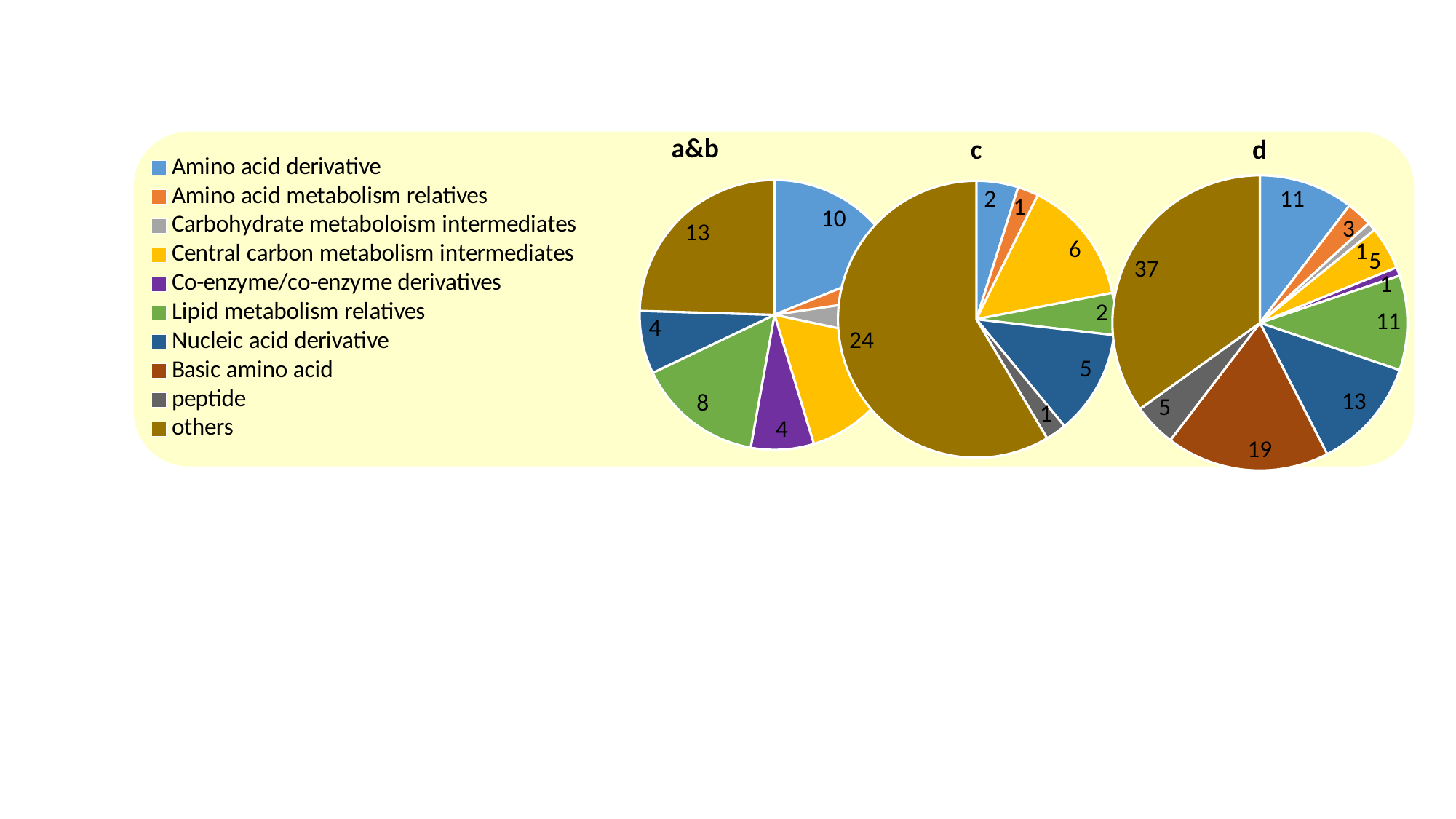

FIGURE S1 | Classification of intestinal metabolites in four different groups displayed in figure 4A. Identified metabolites were classified into ten functional groups (see the text for details).
### Chart: d
| Category | |
|---|---|
| Amino acid derivative | 11.0 |
| Amino acid metabolism relatives | 3.0 |
| Carbohydrate metaboloism intermediates | 1.0 |
| Central carbon metabolism intermediates | 5.0 |
| Co-enzyme/co-enzyme derivatives | 1.0 |
| Lipid metabolism relatives | 11.0 |
| Nucleic acid derivative | 13.0 |
| Basic amino acid | 19.0 |
| peptide | 5.0 |
| others | 37.0 |
### Chart: a&b
| Category | |
|---|---|
| Amino acid derivative | 10.0 |
| Amino acid metabolism relatives | 2.0 |
| Carbohydrate metaboloism intermediates | 3.0 |
| Central carbon metabolism intermediates | 9.0 |
| Co-enzyme/co-enzyme derivatives | 4.0 |
| Lipid metabolism relatives | 8.0 |
| Nucleic acid derivative | 4.0 |
| Basic amino acid | 0.0 |
| peptide | 0.0 |
| others | 13.0 |
### Chart: c
| Category | |
|---|---|
| Amino acid derivative | 2.0 |
| Amino acid metabolism relatives | 1.0 |
| Carbohydrate metaboloism intermediates | 0.0 |
| Central carbon metabolism intermediates | 6.0 |
| Co-enzyme/co-enzyme derivatives | 0.0 |
| Lipid metabolism relatives | 2.0 |
| Nucleic acid derivative | 5.0 |
| Basic amino acid | 0.0 |
| peptide | 1.0 |
| others | 24.0 |
Identified metabolites were classified into ten functional groups from Figure 3A

## Slide 2
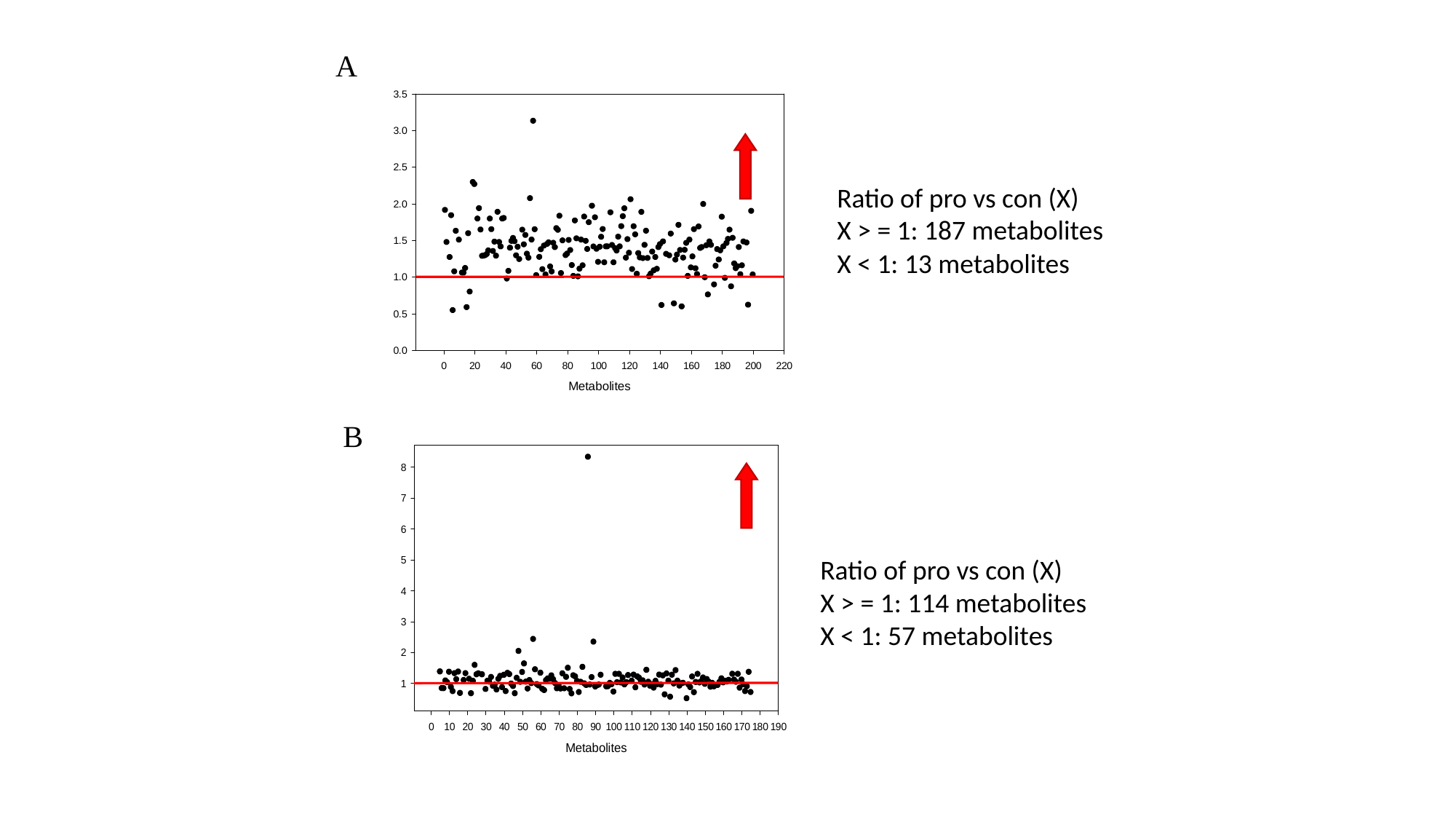

FIGURE S2 | Ratio of metabolite concentration in the probiotic-fed vs control group. (A) Intestinal metabolites; (B) Serum metabolites. The ratio is computed by using averaged detection values.
A
Ratio of pro vs con (X)
X > = 1: 187 metabolites
X < 1: 13 metabolites
B
Ratio of pro vs con (X)
X > = 1: 114 metabolites
X < 1: 57 metabolites

## Slide 3
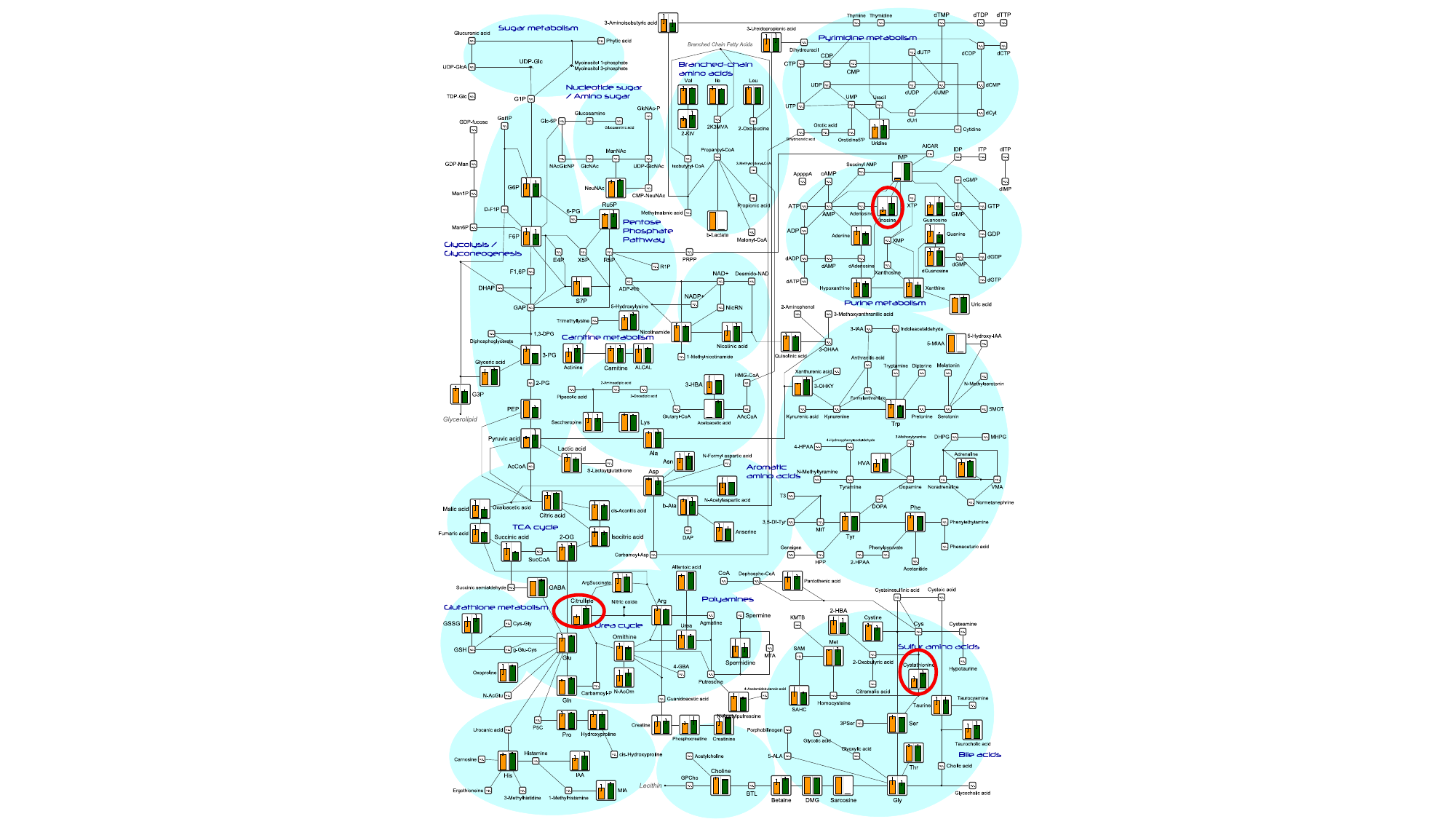

FIGURE S3 | Differences of serum metabolites between control fish and probiotic-fed fish on the systemic metabolic pathways. The relative quantities of the annotated metabolites are represented as bar graphs (green, control; orange, probiotic). Metabolites surrounded by red circles are of higher concentrations in the probiotic group than the control group. ND, not detected.

## Slide 4
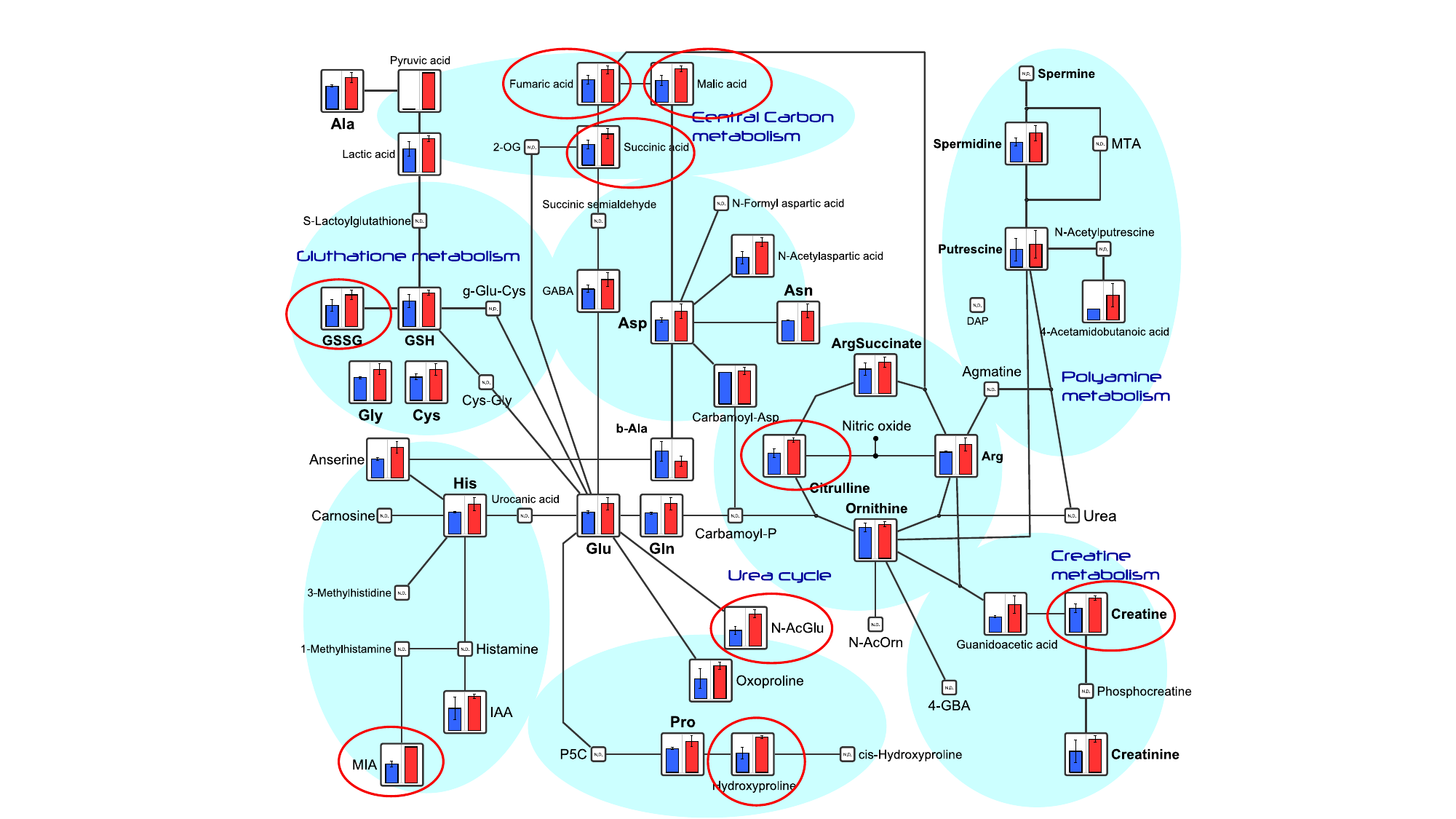

FIGURE S4 | Differences of intestinal metabolites between control fish and probiotic-fed fish on the urea cycle relating metabolism pathways. The relative areas of the annotated metabolites are represented as bar graphs (blue, control; red, probiotic). Metabolites surrounded by red circles are of higher concentrations in the probiotic group than the control group. ND, not detected.

## Slide 5
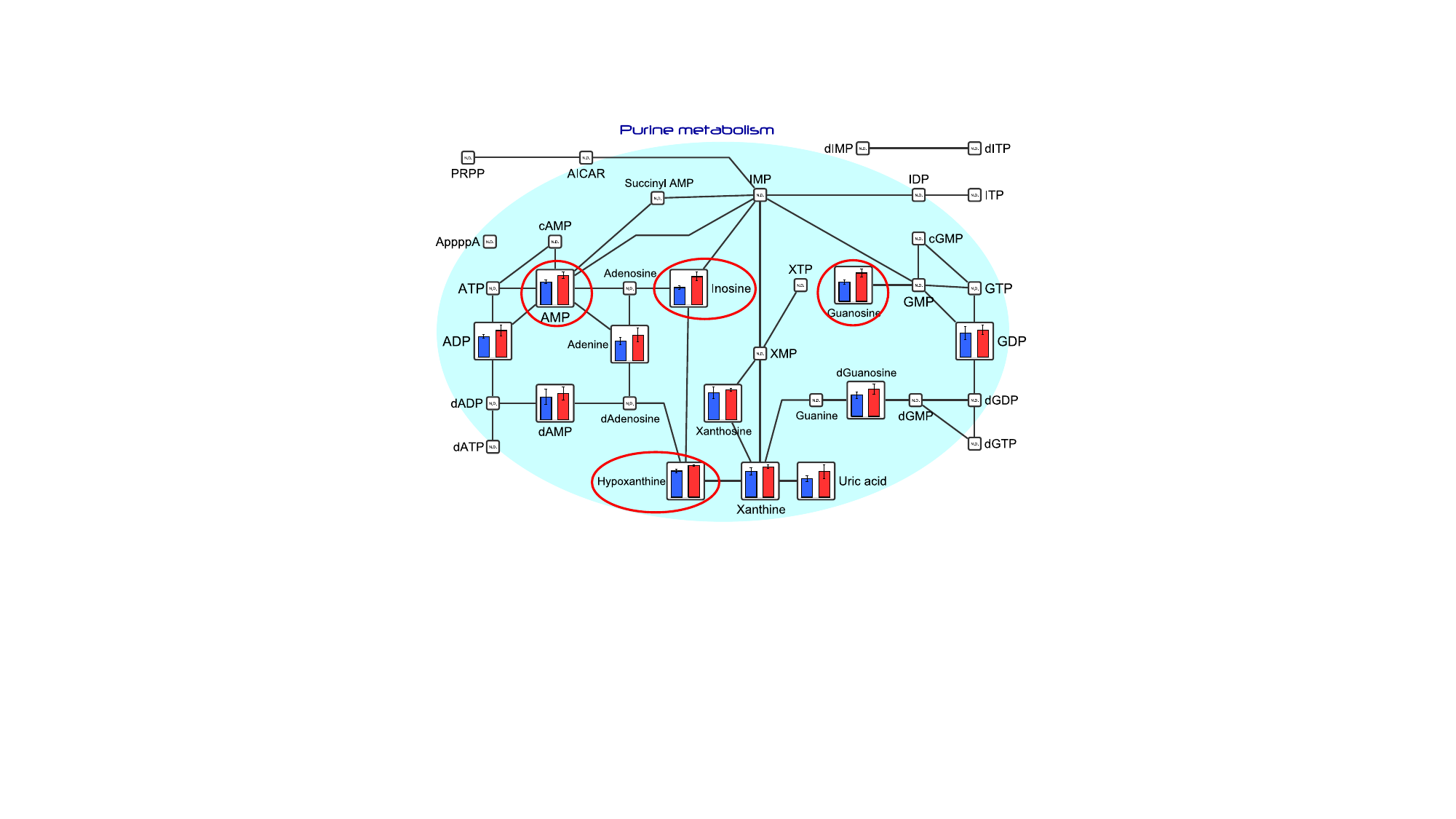

FIGURE S5 | Differences of intestinal metabolites between control and probiotic fish on purine metabolism pathways. The relative quantities of the annotated metabolites are represented as bar graphs (blue, control; red, probiotic). Metabolites surrounded by red circles are of higher concentrations in the probiotic group than the control group. ND, not detected.

## Slide 6
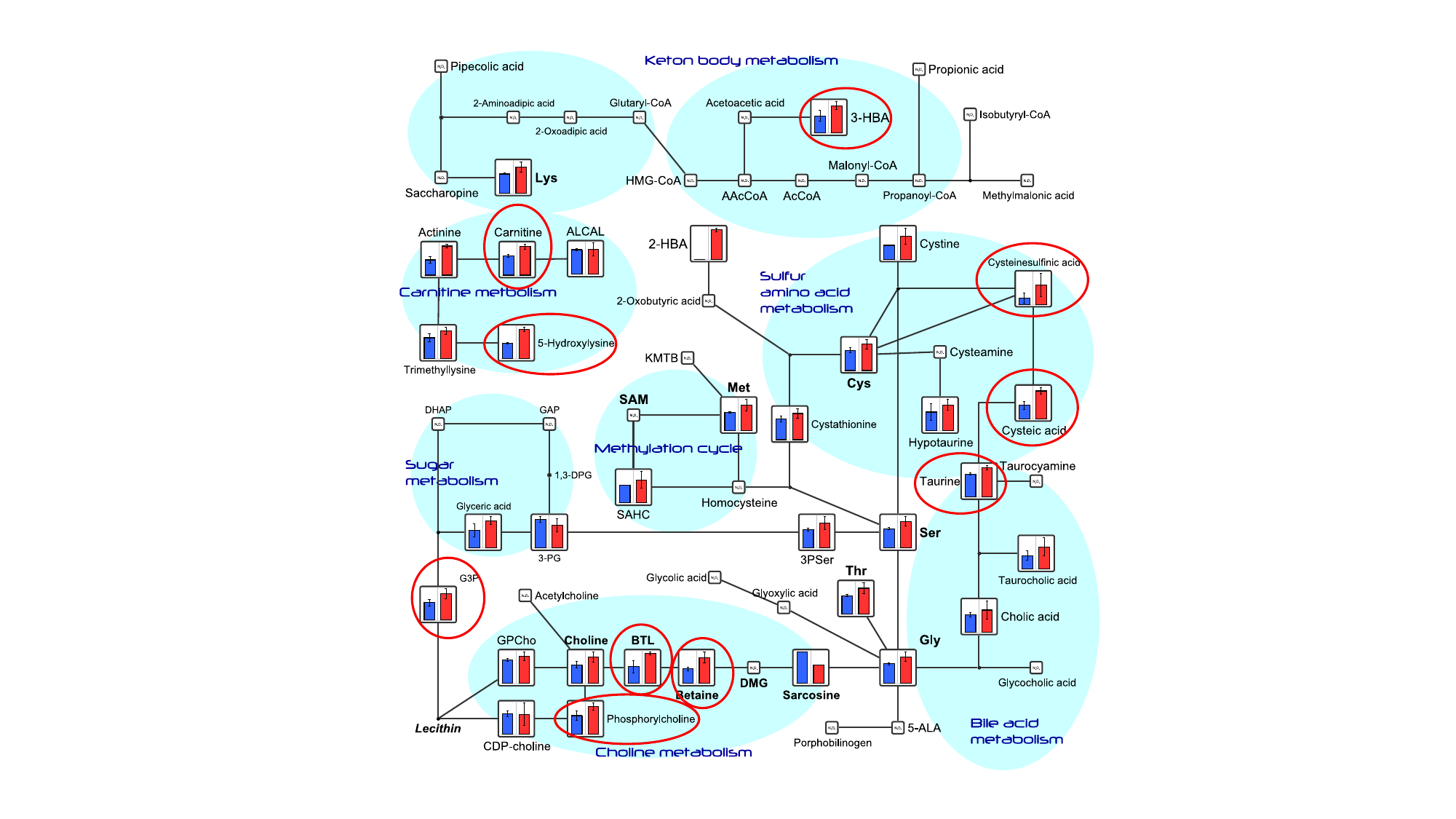

FIGURE S6 | Differences of intestinal metabolites between control fish and probiotic-fed fish on the lipid and amino acid metabolism pathways. The relative areas of the annotated metabolites are represented as bar graphs (blue, control; red, probiotic). Metabolites surrounded by red circles are of higher concentrations in the probiotic group than the control group. ND, not detected.

## Slide 7
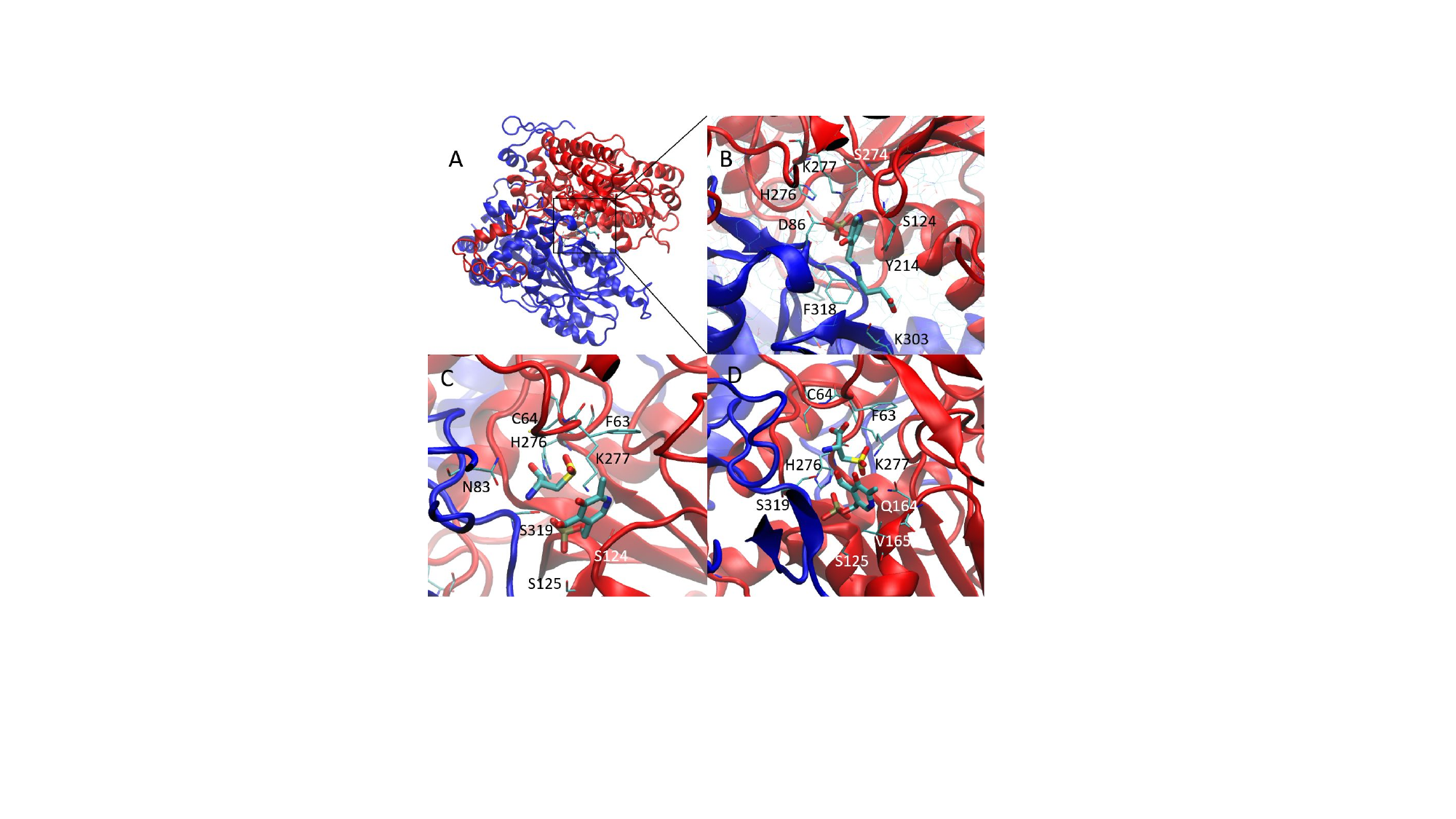

FIGURE S7 | The binding sites of PLP and substrates in L. lactis GAD. The overall dimeric structure of LcGAD is represented by ribbons and colored by blue and red for each chain (A). PLP and substrates (B: L-Glu, C: CSA, D: CA) are represented by thick sticks, and the residues forming the binding site are represented by thin sticks. Carbon, nitrogen, oxygen, phosphorus, and sulfur atoms are colored by cyan, blue, red, gold, and yellow, respectively.

## Slide 8
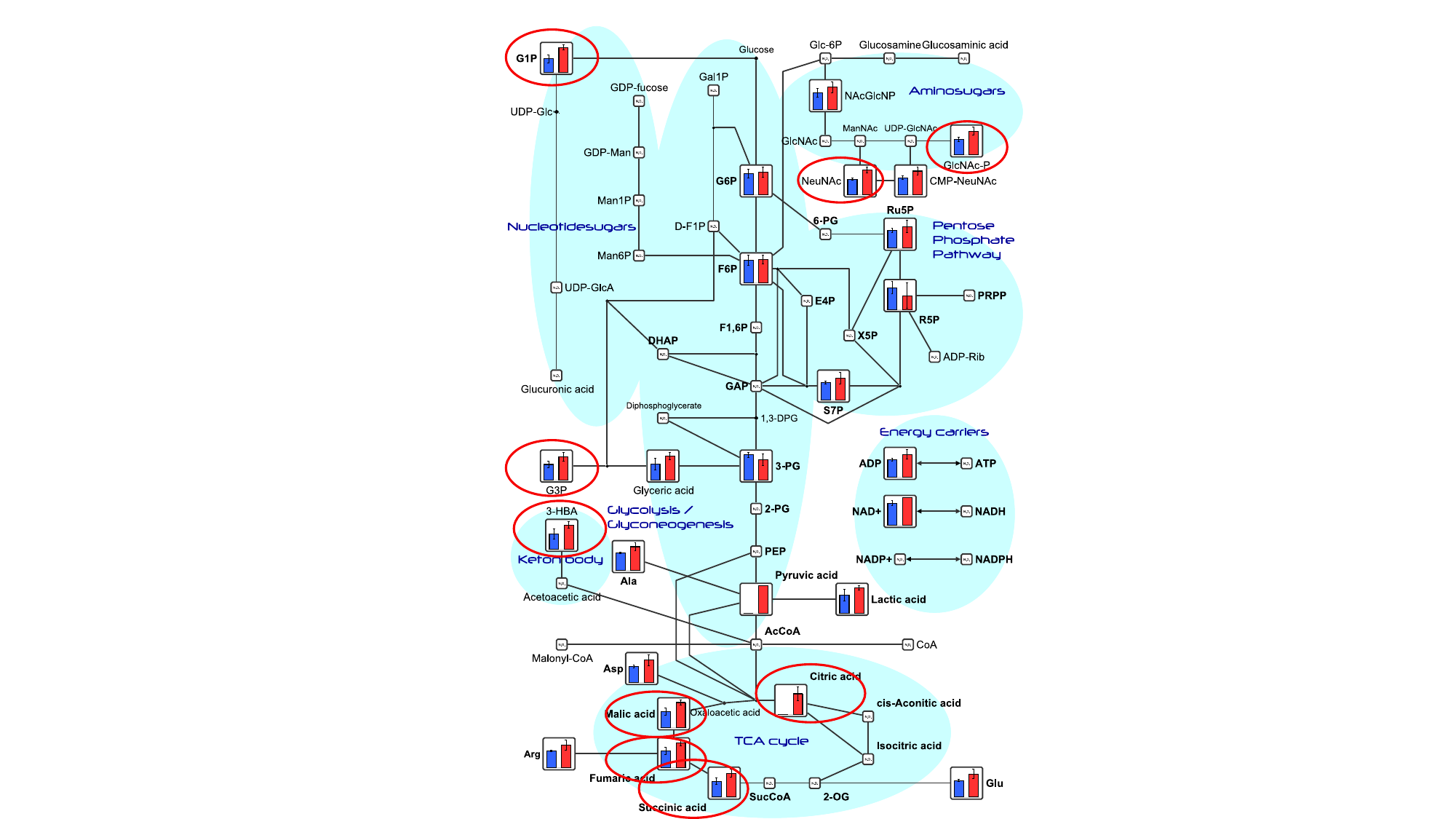

FIGURE S8 | Differences of intestinal metabolites between control fish and probiotic-fed fish on the central carbon metabolism pathways. The relative areas of the annotated metabolites are represented as bar graphs (blue, control; red, probiotic). Metabolites surrounded by red circles are of higher concentrations in the probiotic group than the control group. ND, not detected.
