# Supplementary material for: Dietary Probiotic Effect of Lactococcus lactis WFLU12 on Low-Molecular-Weight Metabolites and Growth of Olive Flounder (Paralichythys olivaceus)
Source: Front Microbiol. 2018 Sep 5;9:2059. doi: 10.3389/fmicb.2018.02059 (PMC6134039; doi:10.3389/fmicb.2018.02059)
Supplement: Table S1 — List of metabolites significantly changed in the serum. 1<, only detected in probiotic group. N.D. (Not Detected): Target peak or metabolite was below detection limits. [file Table_2.DOCX]

**TABLE S1**

| No. | ID | Compound name | Function group | Mean | | SD | | Ratio | |  | Intestine |
| --- | --- | --- | --- | --- | --- | --- | --- | --- | --- | --- | --- |
|  |  |  |  | SerC | SerP | SerC | SerP | SerP vs SerC | *p*-value ^\|\|^ | Co-relation |  |
| 1 | C_0081 | Caffeine | Others | 1.18E-03 | 1.57E-03 | 5.06E-05 | 1.41E-04 | 1.3 | 0.029 | 🡩 | – |
| 2 | C_0088 | Carboxymethyllysine | Amino acid derivative | 1.06E-02 | 1.37E-02 | 9.04E-04 | 1.37E-03 | 1.3 | 0.040 | 🡩 | + |
| 3 | C_0072 | Citrulline | Amino acid derivative | 3.93E-03 | 8.01E-03 | 6.56E-04 | 4.96E-04 | 2.0 | 0.001 | 🡩 | + |
| 4 | C_0092 | Cystathionine | Lipid metabolism relatives | 2.11E-03 | 3.45E-03 | 4.79E-04 | 6.17E-04 | 1.6 | 0.045 | 🡩 | + |
| 5 | A_0053 | Glucuronic acid-1 Galacturonic acid-1 | Carbohydrate metabolism intermediates | 1.06E-04 | 8.80E-05 | 6.62E-06 | 6.94E-06 | 0.8 | 0.031 | 🡫 | + |
| 6 | C_0098 | Homocarnosine | Others | 2.79E-04 | 1.98E-04 | 3.15E-05 | 1.57E-05 | 0.7 | 0.029 | 🡫 | – |
| 7 | C_0073 | Mannosamine | Central carbon metabolism intermediates | 2.35E-04 | 3.05E-04 | 5.58E-06 | 2.65E-05 | 1.3 | 0.039 | 🡩 | + |
| 8 | C_0014 | *N*,*N*-Dimethylglycine | Lipid metabolism relatives | 7.74E-04 | 7.38E-04 | 6.11E-06 | 7.24E-06 | 0.95 | 0.036 | 🡫 | + |
| 9 | A_0083 | Prostaglandin F_2α_ | Lipid metabolism relatives | 1.99E-04 | 1.11E-04 | 2.52E-05 | 3.99E-05 | 0.6 | 0.040 | 🡫 | – |
| 10 | C_0096 | γ-Glu-2-aminobutyric acid | Others | 2.45E-04 | 1.74E-04 | 4.04E-06 | 1.37E-05 | 0.7 | 0.066 | 🡫 | – |
| 11 | A_0036 | Phosphoenolpyruvic acid | Central carbon metabolism intermediates | 1.5E-04 | 9.5E-05 | 3.2E-07 | 8.2E-06 | 0.6 | 0.066 | 🡫 | – |
| 🡩, increase; 🡫, decrease; + present; –, absent | | | | | | | | | | | |

**TABLE S2**

| **No.** | **Compound name** | **Functional group** | **IFP/IFC** |  | **SerP/SerC** | **Category** |
| --- | --- | --- | --- | --- | --- | --- |
| 1 | 3-Phosphoglyceric acid | Central carbon metabolism intermediates | 0.79 | – – –> | 0.67 | i |
| 2 | 3-Aminoisobutyric acid | Amino acid derivative | 0.58 | – – –> | 0.68 | i |
| 3 | Fructose 6-phosphate | Central carbon metabolism intermediates | 1.03 | – – –> | 0.81 | i |
| 4 | Xanthine | Nucleic acid derivative | 1.18 | – – –> | 0.85 | i |
| 5 | Ornithine | Amino acid derivative | 1.11 | – – –> | 0.85 | i |
| 6 | Isethionic acid | Amino acid metabolism relatives | 1.20 | – – –> | 0.89 | i |
| 7 | Lauric acid | Others | 1.20 | – – –> | 0.90 | i |
| 8 | Ethanolamine phosphate | Others | 1.10 | – – –> | 0.92 | i |
| 9 | Glucose 6-phosphate | Central carbon metabolism intermediates | 1.05 | – – –> | 1.00 | i |
| 10 | Terephthalic acid | Others | 1.11 | – – –> | 1.02 | i |
| 11 | O-Acetylcarnitine | Lipid metabolism relatives | 1.00 | – – –> | 1.04 | i |
| 12 | Theobromine | Others | 1.03 | – – –> | 1.05 | i |
| 13 | Cytidine | Nucleic acid derivative | 1.02 | – – –> | 1.10 | i |
| 14 | XA0019 | Others | 0.98 | – – –> | 1.10 | i |
| 15 | Allantoic acid | Others | 1.79 | – – –> | 1.20 | i |
| 16 | Glyceric acid | Lipid metabolism relatives | 1.52 | – – –> | 1.20 | i |
| 17 | Asn | Basic amino acid | 1.47 | – – –> | 1.23 | i |
| 18 | Glucaric acid | Carbohydrate metabolism intermediates | 1.66 | – – –> | 1.25 | i |
| 19 | Guanosine | Nucleic acid derivative | 1.49 | – – –> | 1.25 | i |
| 20 | N-Acetylgalactosamine N-Acetylmannosamine N-Acetylglucosamine | Central carbon metabolism intermediates | 1.26 | – – –> | 1.26 | i |
| 21 | Betaine | Lipid metabolism relatives | 1.79 | – – –> | 1.26 | i |
| 22 | Isoglutamic acid | Others | 1.41 | – – –> | 1.27 | i |
| 23 | Pyruvic acid | Others | 1< | – – –> | 1.27 | i |
| 24 | 5-Oxoproline | Amino acid derivative | 1.64 | – – –> | 1.28 | i |
| 25 | 4-Methyl-2-oxovaleric acid 3-Methyl-2-oxovaleric acid | Others | 1< | – – –> | 1.28 | i |
| 26 | Carboxymethyllysine | Amino acid derivative | 1.53 | – – –> | 1.29 | i |
| 27 | Methionine sulfoxide | Amino acid derivative | 1.69 | – – –> | 1.30 | i |
| 28 | Taurocholic acid | Others | 1.65 | – – –> | 1.30 | i |
| 29 | XC0132 | Others | 1.53 | – – –> | 1.30 | i |
| 30 | XA0033 | Others | 1.46 | – – –> | 1.30 | i |
| 31 | Phosphorylcholine | Lipid metabolism relatives | 1.48 | – – –> | 1.31 | i |
| 32 | Glutathione (GSSG)_divalent | Others | 1.50 | – – –> | 1.31 | i |
| 33 | 5-Hydroxylysine | Amino acid derivative | 1.94 | – – –> | 1.31 | i |
| 34 | 2-Hydroxyvaleric acid | Others | 1< | – – –> | 1.32 | i |
| 35 | Creatinine | Amino acid metabolism relatives | 1.57 | – – –> | 1.36 | i |
| 36 | γ-Butyrobetaine | Amino acid metabolism relatives | 1.90 | – – –> | 1.36 | i |
| 37 | 2-Hydroxy-4-methylvaleric acid | Others | 1.63 | – – –> | 1.36 | i |
| 38 | 1-Methyl-4-imidazoleacetic acid | Others | 1.91 | – – –> | 1.37 | i |
| 39 | Nicotinic acid | Others | 1.63 | – – –> | 1.43 | i |
| 40 | Dyphylline | Others | 1.27 | – – –> | 1.44 | i |
| 41 | Homovanillic acid | Others | 1.41 | – – –> | 1.52 | i |
| 42 | 4-(β-Acetylaminoethyl)imidazole | Others | 2.29 | – – –> | 1.59 | i |
| 43 | Cystathionine | Lipid metabolism relatives | 1.26 | – – –> | 1.63 | i |
| 44 | Citrulline | Amino acid derivative | 1.64 | – – –> | 2.04 | i |
| 45 | Inosine | Nucleic acid derivative | 1.65 | – – –> | 2.34 | i |
| 46 | Threonic acid | Others | 1.99 | →/→ | 1.13 | ii |
| 47 | Sedoheptulose 7-phosphate | Central carbon metabolism intermediates | 1.26 | →/→ | 0.51 | ii |
| 48 | Citraconic acid | Others | 1.24 | →/→ | 0.67 | ii |
| 49 | Succinic acid | Central carbon metabolism intermediates | 1.51 | →/→ | 0.70 | ii |
| 50 | Malic acid | Central carbon metabolism intermediates | 1.55 | →/→ | 0.72 | ii |
| 51 | Butyrylcarnitine | Others | 1< | →/→ | 0.74 | ii |
| 52 | 2-Hydroxyglutaric acid | Others | 1.51 | →/→ | 0.74 | ii |
| 53 | trans-Glutaconic acid | Others | 1.47 | →/→ | 0.74 | ii |
| 54 | Fumaric acid | Central carbon metabolism intermediates | 1.45 | →/→ | 0.77 | ii |
| 55 | Arg-Glu | peptide | 1.48 | →/→ | 0.79 | ii |
| 56 | Glycerol 3-phosphate | Lipid metabolism relatives | 1.51 | →/→ | 0.81 | ii |
| 57 | Adenine | Nucleic acid derivative | 1.31 | →/→ | 0.81 | ii |
| 58 | Cystine | Lipid metabolism relatives | 1.65 | →/→ | 0.82 | ii |
| 59 | Glutaric acid | Carbohydrate metabolism intermediates | 1.29 | →/→ | 0.82 | ii |
| 60 | Gly | Basic amino acid | 1.36 | →/→ | 0.83 | ii |
| 61 | Glucuronic acid-1 Galacturonic acid-1 | Carbohydrate metabolism intermediates | 1.49 | →/→ | 0.83 | ii |
| 62 | 1-Methylhistidine 3-Methylhistidine | Amino acid derivative | 1.47 | →/→ | 0.84 | ii |
| 63 | Asp | Basic amino acid | 1.41 | →/→ | 0.87 | ii |
| 64 | Spermidine | Others | 1.46 | →/→ | 0.87 | ii |
| 65 | 2-Hydroxybutyric acid | Lipid metabolism relatives | 1< | →/→ | 0.87 | ii |
| 66 | Trp | Basic amino acid | 1.48 | →/→ | 0.88 | ii |
| 67 | Lactic acid | Central carbon metabolism intermediates | 1.43 | →/→ | 0.89 | ii |
| 68 | β-Ala | Amino acid derivative | 0.62 | →/→ | 0.90 | ii |
| 69 | Choline | Lipid metabolism relatives | 1.41 | →/→ | 0.90 | ii |
| 70 | Anserine_divalent | Urea cycle relating metabolism | 1.65 | →/→ | 0.91 | ii |
| 71 | S-Adenosylhomocysteine | Lipid metabolism relatives | 1.30 | →/→ | 0.92 | ii |
| 72 | Hypoxanthine | Nucleic acid derivative | 1.20 | →/→ | 0.94 | ii |
| 73 | Phe | Basic amino acid | 1.44 | →/→ | 0.95 | ii |
| 74 | Arg | Basic amino acid | 1.35 | →/→ | 0.95 | ii |
| 75 | Ile | Basic amino acid | 1.41 | →/→ | 0.95 | ii |
| 76 | Nicotinamide | Co-enzyme/co-enzyme derivatives | 1.43 | →/→ | 0.95 | ii |
| 77 | Ser | Basic amino acid | 1.37 | →/→ | 0.95 | ii |
| 78 | Lys | Basic amino acid | 1.36 | →/→ | 0.96 | ii |
| 79 | Ophthalmic acid | Others | 1.27 | →/→ | 0.96 | ii |
| 80 | Ethanolamine | Others | 1.37 | →/→ | 0.96 | ii |
| 81 | Thr | Basic amino acid | 1.40 | →/→ | 0.97 | ii |
| 82 | Carnitine | Lipid metabolism relatives | 1.48 | →/→ | 0.98 | ii |
| 83 | Hydroxyproline | Amino acid derivative | 1.81 | →/→ | 0.99 | ii |
| 84 | SDMA | Amino acid derivative | 1.36 | →/→ | 0.99 | ii |
| 85 | Tyr | Basic amino acid | 1.43 | →/→ | 1.00 | ii |
| 86 | Leu | Basic amino acid | 1.39 | →/→ | 1.00 | ii |
| 87 | Val | Basic amino acid | 1.36 | →/→ | 1.02 | ii |
| 88 | Met | Basic amino acid | 1.41 | →/→ | 1.03 | ii |
| 89 | N-Acetylaspartic acid | Amino acid derivative | 1.93 | →/→ | 1.03 | ii |
| 90 | Taurine | Amino acid metabolism relatives | 1.28 | →/→ | 1.03 | ii |
| 91 | Uric acid | Nucleic acid derivative | 1.38 | →/→ | 1.03 | ii |
| 92 | Creatine | Amino acid derivative | 1.44 | →/→ | 1.04 | ii |
| 93 | Homocitrulline | Amino acid derivative | 1< | →/→ | 1.04 | ii |
| 94 | N6-Acetyllysine | Amino acid derivative | 1.26 | →/→ | 1.04 | ii |
| 95 | Ala | Basic amino acid | 1.36 | →/→ | 1.04 | ii |
| 96 | Cysteine glutathione disulfide | Others | 1.51 | →/→ | 1.04 | ii |
| 97 | Trimethylamine N-oxide | Others | 0.76 | →/→ | 1.05 | ii |
| 98 | XA0012 | Others | 1.41 | →/→ | 1.05 | ii |
| 99 | His | Basic amino acid | 1.38 | →/→ | 1.06 | ii |
| 100 | XA0004 | Others | 1.82 | →/→ | 1.07 | ii |
| 101 | Pantothenic acid | Others | 1.40 | →/→ | 1.07 | ii |
| 102 | 3-Ureidopropionic acid | Nucleic acid derivative | <1 | →/→ | 1.07 | ii |
| 103 | Ribulose 5-phosphate | Central carbon metabolism intermediates | 1.23 | →/→ | 1.07 | ii |
| 104 | N-Acetylneuraminic acid | Central carbon metabolism intermediates | 1.58 | →/→ | 1.07 | ii |
| 105 | Pro | Basic amino acid | 1.31 | →/→ | 1.07 | ii |
| 106 | 2'-Deoxyguanosine | Nucleic acid derivative | 1.27 | →/→ | 1.08 | ii |
| 107 | GABA | Urea cycle relating metabolism | 1.47 | →/→ | 1.09 | ii |
| 108 | 3-Hydroxybutyric acid | Central carbon metabolism / Lipid and amino acid metabolism | 1.59 | →/→ | 1.10 | ii |
| 109 | XA0036 Ascorbate 2-sulfate | Others | 1.52 | →/→ | 1.11 | ii |
| 110 | Gluconic acid | Central carbon metabolism intermediates | 1.64 | →/→ | 1.12 | ii |
| 111 | Argininosuccinic acid | Amino acid derivative | 1.29 | →/→ | 1.13 | ii |
| 112 | Glu | Basic amino acid | 1.40 | →/→ | 1.14 | ii |
| 113 | Gln | Basic amino acid | 1.46 | →/→ | 1.15 | ii |
| 114 | Uridine | Nucleic acid derivative | 1.23 | →/→ | 1.15 | ii |
| 115 | Citric acid | Central carbon metabolism intermediates | 1< | →/→ | 1.17 | ii |
| 116 | Thiamine | Co-enzyme/co-enzyme derivatives | 1.69 | →/→ | 1.18 | ii |
| 117 | Imidazole-4-acetic acid | Urea cycle relating metabolism | 1.54 | →/→ | 1.19 | ii |
| 118 | Stachydrine | Amino acid metabolism relatives | 1.01 | →/→ | 1.21 | ii |
| 119 | Glycerol | Others | 1.00 | →/→ | 1.49 | ii |
| 120 | Diethanolamine | Others | <1 | →/→ | 2.42 | ii |
| 121 | Sarcosine | Lipid metabolism relatives | 0.59 | →/→ | <1 | ii |
| 122 | Itaconic acid | Others | 1.88 | →/→ | <1 | ii |
| ##, Transportation state predicted following a previous study (Matsumoto et al., 2017) | | | | | |  |
| – – –>, Metabolites may be transported without any influences by the gateways;  →/→, Metabolites may be influenced by the gateways. | | | | | |  |
| Metabolites detected only in the probiotic-fed or the control fish are indicated by 1< or <1, respectively. | | | | | |  |

**TABLE S3**

|  | | | | |
| --- | --- | --- | --- | --- |
| **Substrates** | **Chain A** | | **Chain B** | |
|  | **PLP** | **Substrate** | **PLP** | **Substrate** |
| **L-Glu** | Asp86, Phe318 | Lys303 | Ser124, Ser274, His276, Lys277 | Tyr214 |
| **CSA** | Ser319 | Asn83, Ser319 | Ser124, Ser125, Lys277 | Phe63, Cys64, His276, Lys277 |
| **CA** | Ser319 | Ser319 | Ser125, Gln164, Val165, His276 | Phe63, Cys64, Lys277 |
