# Supplementary material for: Dietary Probiotic Effect of Lactococcus lactis WFLU12 on Low-Molecular-Weight Metabolites and Growth of Olive Flounder (Paralichythys olivaceus)
Source: Front Microbiol. 2018 Sep 5;9:2059. doi: 10.3389/fmicb.2018.02059 (PMC6134039; doi:10.3389/fmicb.2018.02059)
Supplement: Supplementary file 4 [file Data_Sheet_1.docx]

**Dietary probiotic effect of *Lactococcus lactis* WFLU12 on low-molecular-weight metabolites and growth of olive flounder (*Paralichythys olivaceus*)**

Thanh Luan Nguyen*^1,2^*, Won-Kyong Chun*^2^*, Ahran Kim*^2^*, Nameun Kim*^2^*, Heyong Jin Roh*^2^*, Yoonhang Lee*^2^*, Myunggi Yi*^3^*, Suhkmann Kim*^4^*, Chan-Il Park*^5,^**, Do-Hyung Kim*^2,^**

*^1^Department of Veterinary Medicine, HUTECH Institute of Applied Science, Ho Chi Minh City University of Technology, Ho Chi Minh City 700000, Vietnam. E-mail: nt.luan@hutech.edu.vn*

*^2^Department of Aquatic Life Medicine, College of Fisheries Science, Pukyong National University, Busan, Republic of Korea*

*^3^Department of Biomedical Engineering, College of Engineering, Pukyong National University, Busan 48513, Republic of Korea*

*^4^Department of Chemistry, Center for Proteome Biophysics and Chemistry Institute for Functional*

*Materials, Pusan National University, Busan, 46241, Republic of Korea*

*^5^Department of Marine Biology & Aquaculture, College of Marine Science, Gyeongsang National University, 455, Tongyeong 650-160, Republic of Korea*

***Corresponding author: Fax:** +82 51 6295877

*E-mail address:* [vinus96@hanmail.net](mailto:vinus96@hanmail.net) (C.-I. Park), [dhkim@pknu.ac.kr](mailto:dhkim@pknu.ac.kr) (D.-H. Kim)

**Supplementary information include:**

Supplementary materials and methods of CE-TOFMS analysis

Supplementary information for molecular modeling of *L. lactis gad*

**Supplementary materials and method of CE-TOFMS analysis**

*Weight measurement and sampling*

Initial weights of all olive flounder for each aquarium were determined. Weight and length of these fish were then measured once every four weeks. Feed ration was then adjusted accordingly. Weight gain was calculated as the difference between final and initial biomass. Specific growth rate (SGR) was calculated using the following formula: [ln (Final weight) – ln (Initial weight)/days] × 100. Feed efficiency (FE) was calculated as weight gain (g) / feed intake (g). At the end of the feeding experiment (16 weeks), nine fish were collected from each experimental group and weight and length were measured. The serum and intestinal mucus were used for ionic metabolites analyses by CE-TOFMS. Intestinal mucus was collected, weighted, and homogenized with cold phosphate buffered saline (PBS, pH 7.5). Enumeration of probiotic bacteria was performed after plating and incubation of serially diluted mucus homogenate on selective MRS medium at 28^o^C for 48 h.

*Preparation of intestinal fluid, serum, and pellet for metabolome analysis*

Following anesthesia, blood (3 ml) was first collected through the caudal vein using a syringe. Blood was incubated at 4^o^C for 20 min and serum was harvested by centrifugation. After blood collection, intestines were aseptically excised and separated. Intestinal mucus (approximately 0.5 g) were suspended in 500 μL of cold PBS (pH 7.5) to yield final volume of 1.0 mL followed by homogenization using a homogenizer. The homogenate was then centrifuged at 15000×g for 15 min at 4^o^C. Aliquots of serum and intestinal fluid samples were stored at −80^o^C until analysis. Feed pellets were crushed by mortar and prepared for analysis.

For CE-TOFMS analyses of ionic metabolites present in fish intestinal fluids and serum samples, each sample (50 µL) was mixed with 450 µL of methanol containing internal standards (10 µM). Chloroform (500 µL) and Milli-Q water (200 µL) were then added, mixed thoroughly, and centrifuged (2,300 x g, 4˚C, 5 min). The water layer (400 µL × 1) was filtrated through 5-kDa cut-off filter (ULTRAFREE-MC-PLHCC, Human Metabolome Technologies, Yamagata, Japan) to remove macromolecules. The filtrate was then centrifugally concentrated and re-suspended in 25 µL of ultrapure water immediately.

For analyzing ionic compounds present in fish feed pellet by CE-TOFMS, the pellet (approximately 20 mg) supplemented with or without probiotic was crushed by mortar and then mixed with 600 µL of methanol containing internal standards (50 µM) and homogenized with a homogenizer (1,500 rpm, 120 sec × 2 times). Chloroform (600 µL) and Milli-Q water (240 µL) were then added to the homogenate, mixed thoroughly, and centrifuged at 2,300 x g for 5min at 4˚C. The water layer (200 µL × 2) was filtrated through 5-kDa cut-off filter (ULTRAFREE-MC-PLHCC, Human Metabolome Technologies-HMT, Yamagata, Japan) to remove macromolecules. The filtrate was then centrifugally concentrated and re-suspended in 50 µL of ultrapure water immediately.

*CE-TOFMS*

Extracted metabolites were measured in Cation and Anion modes using Agilent CE-TOFMS system (Agilent Technologies Inc., Waldbronn, Germany) for metabolome analysis using conditions described previously (Soga and Heiger, 2000; Soga et al., 2002; Soga et al., 2003). These samples were diluted for measurement. To improve analysis qualities of CE-MS, dilution factors of 50 and 10 were used for cation and anion modes, respectively.

For cationic and anionic metabolites, capillary electrophoreses were performed using a fused silica capillary with inner diameter and total length of 50 mm and 80 cm, respectively. Measurement of compounds in both positive and negative modes was performed using commercial electrophoresis buffer (Solution ID H3301-1001 for cation mode and H3302-1021 for anion mode; HMT Inc., Tsuruoka, Japan). Pressure of 50 mbar was applied to inlet capillary during run for 10 seconds in cation mode and 25 seconds in anion mode. Electrospray ionization TOF-MS was operated in positive ion mode (4,000 V) and negative ion mode (3,500 V) for cationic metabolites and anionic metabolites, respectively. Peaks detected in CE-TOFMS analysis were extracted using automatic integration software (MasterHands ver. 2.16.0.15 developed at Keio University) to obtain peak information, including m/z, migration time (MT), and peak area. Exact mass data were acquired over a range of 50–1000. Alignment of detected peaks was performed according to m/z value and normalized migration time. Finally, peak areas were normalized against those of internal standards MetSul and Dcamphor-10-sulfonic acid (CSA) for cationic and anionic metabolites, respectively. Annotation tables were produced from measurement of standard compounds and aligned with datasets according to similar m/z value and normalized migration time.

**Supplementary information for molecular modeling of *L. lactis* *gad***

**Materials and methods**

In order to predict whether *L. lactis* glutamic acid decarboxylase GAD (LcGAD), an enzyme involved in mechanisms for pH homeostasis by consuming intracellular protons from a decarboxylation process, has detectable activity to glutamic acid, CSA or CA, and to elucidate the structural basis of LcGAD substrate selectivity, its structural model was generated by SWISS-MODEL (Biasini et al., 2014) using the atomic coordinates of Escherichia coli GAD (EcGAD, PDB: 1PMM) as a template. LcGAD shares 45.39% sequence identity with EcGAD (Figure S7). The LcGAD structural model was then used to assess the potential structural basis of its substrate binding through molecular dynamics (MD) simulations. Simulations were performed using Gromacs 5.0.4 (Van der Spoel et al., 2005) with Gromos54a7 force fields (Schmid et al., 2011) for protein, water and ions, and the atomic charges and topology files (force fields) for PLP (pyridoxal-5’-phosphate as a cofactor) and substrate molecules (L-Glu, CSA, and CA) were developed by ATB (Koziara et al., 2014).

The structures of homology model of the enzyme and PLP with the substrates were further relaxed using MD simulations under physiological conditions to evaluate the interactions between the substrates and the enzyme. The enzyme with each substrate was solvated in a dodecahedron box filled with SPC water molecules (Berweger et al., 1995). All three simulation systems, then, were electrically neutralized with Na+ and Cl- ions in a concentration of 0.15 M. Energy minimization was performed to remove bad contacts within 5000 steps. Then the systems were gradually heated up to 300 K under constant volume condition in a period of 60 ps. In order to equilibrate the density, systems were subjected to constant-pressure (1 bar) and constant-temperature (300 K) conditions (NPT ensemble) for 1.0 ns. Then simulations continued to the production stage for ~260 ns under the same condition.

**Results**

The potential binding sites of PLP with three substrates are illustrated in Figure S7 and summarized in Table S3. In the simulation of L-Glu system, PLP made hydrogen bond interactions with side chains of Asp86, Ser274, His276, and Lys277 and with backbone of Ser124. The substrate, L-Glu, made hydrogen bond with side chain of Tyr124 and backbone of Lys303. In CSA system, PLP made hydrogen bond with side chains of Ser125, Ser319, Lys277 and with backbone of Ser124, Ser125. CSA made hydrogen bond with side chain of Cys64, Asn83, His276, Lys277, Ser319 and backbone of Phe63 and Cys64. In CA system, PLP made hydrogen bond with side chains of Ser125, Gln164, His276, Ser319 and with backbone of Ser125 and Val165. CA made hydrogen bond with side chain of Cys64, Lys277, Ser319 and backbone of Phe63, Cys64. Unlike L-Glu, CSA and CA simulation showed that their binding sites were shared. Due to the negatively charged PLP and substrate molecules, they were mainly electrostatic interaction between the ligands and the enzyme. Also, the polar or charged amino acid residues of binding sites indicate the electrostatic interaction are the major source of attraction in these complexes.

**References**

Berweger, C.D., van Gunsteren, W.F. and Müller-Plathe F. (1995). Force field parametrization by weak coupling: re-engineering SPC water. *Chem.* *Phys. Lett.* 232, 429-36. doi: 10.1016/0009-2614(94)01391-8

Biasini, M., Bienert S., Waterhouse A., Arnold K., Studer G., Schmidt T., Kiefer F., Gallo Cassarino T., Bertoni M., Bordoli L., Schwede T. (2014). SWISS-MODEL: modelling protein tertiary and quaternary structure using evolutionary information. *Nucleic Acids Res.* 42, W252–W258. doi: 10.1093/nar/gku340

Koziara, K.B., Stroet, M., Malde, A.K., Mark A.E. (2014). Testing and validation of the Automated Topology Builder (ATB) version 2.0: prediction of hydration free enthalpies. *J. Comput. Aided. Mol. Des.* 28(3), 221-33. doi: 10.1007/s10822-014-9713-7

Schmid N., Eichenberger A.P., Choutko A., Riniker S., Winger M., Mark A.E., van Gunsteren W.F., (2011). Definition and testing of the GROMOS force-field versions 54A7 and 54B7. *Eur. Biophys. J.* 40(7), 843-856. doi: 10.1007/s00249-011-0700-9

Soga, T., Heiger, D.N. (2000). Amino acid analysis by capillary electrophoresis electrospray ionization mass spectrometry. *Anal.Chem.* 72: 1236-1241. doi: 10.1021/ac990976y

Soga, T., Ohashi, Y., Ueno, Y., Naraoka, H., Tomita, M., Nishioka T. (2003). Quantitative metabolome analysis using capillary electrophoresis mass spectrometry. *J. Proteome Res.* 2, 488-494. doi: 10.1021/pr034020m

Soga, T., Ueno, Y., Naraoka, H., Ohashi, Y., Tomita, M., Nishioka T. (2002). Simultaneous determination of anionic intermediates for *Bacillus subtilis* metabolic pathways by capillary electrophoresis electrospray ionization mass spectrometry. *Anal.Chem.* 74: 2233-2239, 2002. doi: 10.1021/ac020064n

Van der Spoel, D., Lindahl, E., Hess, B., Groenhof, G., Mark, A. E., Berendsen, H. J. C. Gromacs: fast, flexible, and free. *J. Comput. Chem.* 2005, 26 (16), 1701–1718. doi:10.1002/jcc.20291
